# Recombinant structures expand and contract inter and intragenic diversification at the KIR locus

**DOI:** 10.1186/1471-2164-14-89

**Published:** 2013-02-08

**Authors:** Chul-Woo Pyo, Ruihan Wang, Quyen Vu, Nezih Cereb, Soo Young Yang, Fuh-Mei Duh, Steven Wolinsky, Maureen P Martin, Mary Carrington, Daniel E Geraghty

**Affiliations:** 1 Clinical Research Division, Fred Hutchinson Cancer Research Center, Seattle, WA, USA; 2 Histogenetics LLC, Ossining, NY, USA; 3 Cancer and Inflammation Program, Laboratory of Experimental Immunology, Frederick National Laboratory for Cancer Research, Frederick, MD, USA; 4 Division of Infectious Diseases, The Feinberg School of Medicine, Northwestern University, Chicago, IL, USA

**Keywords:** Natural killer cells, Human, KIR, Recombinant structures

## Abstract

**Background:**

The human *KIR* genes are arranged in at least six major gene-content haplotypes, all of which are combinations of four centromeric and two telomeric motifs. Several less frequent or minor haplotypes also exist, including insertions, deletions, and hybridization of *KIR* genes derived from the major haplotypes. These haplotype structures and their concomitant linkage disequilibrium among *KIR* genes suggest that more meaningful correlative data from studies of *KIR* genetics and complex disease may be achieved by measuring haplotypes of the *KIR* region in total.

**Results:**

Towards that end, we developed a KIR haplotyping method that reports unambiguous combinations of *KIR* gene-content haplotypes, including both phase and copy number for each *KIR*. A total of 37 different gene content haplotypes were detected from 4,512 individuals and new sequence data was derived from haplotypes where the detailed structure was not previously available.

**Conclusions:**

These new structures suggest a number of specific recombinant events during the course of *KIR* evolution, and add to an expanding diversity of potential new KIR haplotypes derived from gene duplication, deletion, and hybridization.

## Background

Natural killer (NK) cells are lymphocytes that act as central components of the innate immune response, providing immediate defense against infectious agents [1]. As part of the mechanisms directing this function, these cells utilize a number of stimulatory and inhibitory receptors that react with major histocompatibility complex (MHC) class I antigens expressed by host cells. These NK receptors include variable killer cell immunoglobulin-like receptors (KIR) that in humans interact with the polymorphic HLA-A, B, and C ligands [2] and the nearly invariant CD94/NKG2 family of receptors that interact with the conserved HLA-E ligand [3,4]. For *KIR*, variation can be described at two levels, gene content and allelic variation. Early reports of gene content variation described two major haplotypes: group A, consisting of inhibitory KIR and one noninhibitory KIR; and group B, containing additional inhibitory and noninhibitory KIR not found in group A [5]. The genomic structure of the KIR region has been extensively studied [6-8] and the complete genomic sequence of 24 *KIR* haplotypes has been delineated [9].

The genetic variation of *KIR* and the central role of NK cells in the immune response – including infectious disease and tumor immunity – have spurred investigation into the association of *KIR* genetics with a number of diseases and immunologic responses. Many of these studies have focused on the presence or absence of *KIR* genes and association with infectious disease [10]. For example, the presence of KIR2DL3 and its HLA-C1 ligand directly influenced resolution of hepatitis C virus infection [11] and herpes simplex type 1 infection was modified by the high-affinity receptor/ligand pair KIR2DL2/HLA-C1 [12]. Further, both genetic and functional data point to interactions between specific *KIR* genes and their HLA ligands in managing HIV infection [13]. On the dysfunctional side of the immune response, several autoimmune diseases appear to be associated with the presence of a subset of activatory *KIR* genes, including *KIR2DS2,* which is associated with rhuematoid arthritis [14], and scleroderma [15]. *KIR* was implicated in hematopoetic cell transplantation not long after the delineation of *KIR* genetic structures [16] and more recently specific KIR haplotype substructures were associated with improved outcomes of unrelated transplants for acute myelogenous leukemia [17]. Finally, KIR receptors and HLA ligands have been implicated in normal processes of pregnancy where allorecognition of paternal HLA-C by maternal KIR is postulated to be involved in essential trophoblast invasion and vascular remodeling and in turn potential derivative complications of pregnancy [18].

Our previous study of the *KIR* region yielded detailed information about *KIR* haplotype structures derived uniquely from complete phased genomic sequences, revealing substructures of known haplotypes and further defined linkage among the *KIR* genes. Taking into account the many reported correlations between *KIR* polymorphism and disease, there is some imperative to incorporate KIR haplotype structures, including potential intraregion epistasis, into the association studies so that causative variation at *KIR* can be identified. In addition, the repetitive gene content structure of the *KIR* region may contribute to rapid evolution through aberrant recombination mechanisms, possibly driven by the immune function of KIR and providing further impetus towards understanding overall genomic region variation. Towards these ends, we set out to establish methods for genotyping *KIR* that would yield the structures of phased haplotypes as defined by gene content. In the course of this development, we uncovered a number of new haplotype substructures and further defined genomic sequences that account for a large majority of those found in human populations. Stemming from the establishment of a comprehensive genotyping/haplotyping methodology, the results suggested that a number of different recombination events intertwined with strong linkage disequilibrium have been directly involved in the expansion and contraction of the *KIR* locus.

## Methods

### Cell lines/source DNAs

The DNAs used for library construction were extracted from a panel of cell lines chosen based on results of genotyping that indicated novel arrangements of KIR loci. DNA was prepared from B-LCLs using a Qiagen (Valencia, CA) genomic DNA extraction kit according to the manufacturers instructions. DNAs were obtained from the Research Cell Bank (RCB) at the Fred Hutchinson Cancer Research Center and are commercially available (http://www.ihwg.org). DNAs used in genotyping were derived from four sources, (1) a panel of 1,500 allogeneic hematopoietic cell transplantation (HCT) donors and recipients enrolled in a genome-wide association study [19] and other reference DNAs from the IHWG.org totaling 3,229 samples, (2) a subset of the STEP HIV vaccine trials study samples described [20] totaling 935, (3) DNAs from the UCLA DNA exchange for HLA typing, totaling 144 and (4) DNAs from the Carrington laboratory totaling 174. The ethnicity of these DNAs is approximately 85% Caucasian, 10% Asian, and 5% African and Hispanic. Data for finer breakdown of these ethnicities was not available, however a listing of haplotype frequencies for a panel of four ethnic groups used in this study is included in supplementary Additional file 1: Table S3.

### KIR gene content haplotyping

Fourteen amplicons and the primary and secondary SNP positions within each that can be used for the detection of individual KIR genes are listed in Table 1 and rules for gene copy variation are listed in Table 2. PCR-sequencing to generate sequence traces for haplotype mapping was carried out according to established procedures [21,22]. All data from PCR-sequencing assays were automatically interpreted by software developed in house (heterozygous-trace resolution or HTR), which accurately interprets dye-terminator sequence traces from heterozygous DNAs [22,23]. Specialized scripts were developed to assemble knowledge from all 14 assays to detect gene copy numbers including validation of concordant data (and reporting of discordant data), logically match with consistent pairs of known haplotypes, and report details on all experimental variables examined.

**Table 1:** KIR haplotyping methodology – primary and secondary SNP positions

**Amplicon**^**a**^	**Exon**	**Genes amplified**^b^	**Primary validation**^**c**^	**Secondary confirmation**^**d**^
**2DL2-001**	5	2DL2, 2DL3, 2DS2, 2DP1, (3DL2)	2DL2-93-T	2DP1-137-T/2DS2-209-T
**2DL3-006**	7/8	2DL3, 2DS3/5	2DL3-136-G; -616-T	2DS35-59-A
**2DL5**	3	2DL1/4/5 3DP1	2DL5-170-G; -174-A	2DL5-58-G; -79-T; -103-C; -157-A
**2DP1-002**	3	2DP1	2DP1-128-G; -130-A	
**2DS2-002**	4	2DL1/2/3, 2DS2, 3DP1	2DS2-89-A	2DL1-60-A; -117-A;-154-A
**2DS35-005**	4	2DS1, 2DS3/5, 3DP1, (2DL1)	2DS35-247-T; -251-G; -403-A; -404-T2DS3-446-G; -507-T2DS5-376-C	2DS1-446-A
**3DL1-002**	4	3DL1, 3DS1	3DL1-85-T3DS1-85-G	
**3DL1-005**	4	3DL1, 3DS1	3DL1-157-C; -167-T3DS1-157-T; -167-G	
**KIR-2DL2-N1**	4	2DL2/3, 2DS2, 2DP1	2DL2-126-G	2DS2-213-A2DP1-217-C
**KIR-2DS2-N1**	5	2DL2/3 2DP1 2DS2	2DS2-92-T	2DL2-179-T
**2DS1-4-002**	4	2DS1, 2DL1, 2DS4 (2DS35)	2DS1-257-AG2DL1-257-C2DS4-86-T; -95-A	2DS4-76-T; -86-T; -138-G; -187-A; -95-A; -179-A; -197-A; -225-T; -246-T; -345-G
**2DS1-004**	4	2DS1, 2DL1, (2DS4)	2DS1-80-AG2DL1-80-C	2DL1-139-G2DS1-139-T
**3DL1-3DS1-01**	4	3DL1, 3DS1, 3DL2	multiple SNPs^e^	
**3DL1-3DS1-05**	4	3DL1, 3DS1, 3DL2	multiple SNPs^e^	

^a^ see supplementary Additional file 1: Table S3 for primer sequences.

^b^ loci listed in parenthesis indicate secondary PCR products amplified only in the absence of the target locus.

^c^ The position(s) of SNP(s) within the amplicon uniquely identifying the target locus.

^d^ The position(s) of SNP(s) which identify other loci amplified by that assay.

^e^ multiple SNP positions indicate presence or absence of 3DL1/S1.

**Table 2:** KIR haplotyping methodology – SNP ratios for gene copy number

			**Gene copy determination using peak ratios**		
**Amplicon**^a^	**Exon**	**Genes amplified**^b^	**Gene ratios**	**SNP ratios**^c^	**Observed peak ratios**
**2DL2-001**	5	2DL2, 2DL3, 2DS2, 2DP1 (3DL2)	(2DS2+2DL2+2DL3):2DP1	C:T at 137	1:1, 2:1, 3:1, 3:2, or 4:1
			(2DL2+2DL3):2DP1	A:C at 206	1:1 or 2:1
			(2DL2+2DL3):2DS2	206(A):209(T)	1:1 or 2:1
**2DL3-006**	7/8	2DL3, 2DS3/5	2DL3:2DS35	G:T at 136	1:1, 2:1, 1:2
**2DL5**	3	2DL1/4/5 3DP1	2DL5:(2DL4+3DP1)	G:C at 170	1 or multiple copies
**2DP1-002**	3	2DP1	No ratio data		
**2DS2-002**	4	2DL1/2/3, 2DS2, 3DP1	No ratio data		
**2DS35-005**	4	2DS1, 2DS3/5, 3DP1, (2DL1)	2DS1:2DS3	A:G at 446	1:1 or 1:2
			2DS3:2DS5	446(G):447(A)	1:1, 2:1, 1:2
**3DL1-002**	4	3DL1, 3DS1			
**3DL1-005**	4	3DL1, 3DS1	3DL1:3DS1	T:G at 167	1:1
**KIR-2DL2-N1**	4	2DL2/3, 2DS2 2DP1	2DS2:2DP1	213(A):217(C)	1:1 or 1:2
**KIR-2DS2-N1**	5	2DL2/3 2DP1 2DS2	No ratio data		
**2DS1-4-002**	4	2DS1, 2DL1, 2DS4 (2DS35)	2DS1:2DL1:2DS4	A:C:T at 257	1:1:1, 1:2:1, 0:1:1, 0:2:1, or 0:1:2
**2DS1-004**	4	2DS1, 2DL1, (2DS4)	No ratio data		
**3DL1-3DS1-01**	4	3DL1, 3DS1, 3DL2	(3DL1+3DS1):3DL2	Multiple SNPs	1:1, 0:2, 1:2 or 3:2
**3DL1-3DS1-05**	4	3DL1, 3DS1, 3DL2	(3DL1+3DS1):3DL2	Multiple SNPs	1:1, 0:2, 1:2 or 3:2

^a^ see supplementary Additional file 1: Table S3 for primer sequences.

^b^ loci listed in parenthesis indicate secondary PCR products amplified only in the absence of the target locus.

^c^ SNPs in parenthesis indicate ratios determined on nonoverlapping positions.

### Genomic DNA sequencing

Long-range PCR and fosmid clones were used to isolate phased genomic regions. Fosmid library construction and isolation was carried out as described in Raymond *et al*. [24] with modifications [9]. Fosmid libraries were produced from five cell lines and long-range PCR products from 3 additional DNAs as depicted in Additional file 2: Figure S1. Briefly, fosmid library constructions used the Epicentre copy-control vector pCC1. Sheared, end-repaired inserts were size-selected to be 30–50 Kbp by pulsed-field-gel electrophoresis. Packaging was carried out with the Epicentre MaxPlax extracts and transfection was into Epicentre EPI300-T1^R^*E. coli* cells. Lysed aliquots of induced cultures were used directly as a source of PCR template during the STS-content mapping of 3,000-fosmid pools and at all subsequent stages of screening. For KIR screening, a panel of unlabeled primers designed locally and ordered from Sigma-Genosys were used to amplify each of the known KIR genes. Additional file 1: Table S1 includes the sequences and gene specificity for the primer pairs used for all of the fosmid screening carried out and the copy number validation in some cases when the information of gene copy number was required. Samples were scored as positive or negative for a particular PCR assay based on SYBR green fluorescence (ABI). Data was collected on an ABI 7900 instrument operating in real-time (not end-point) mode.

All long-range PCRs were done using Platinum Taq High Fidelity (Invitrogen, Life Technologies Corporation). Reagents were added in the following order: 10 mM dNTPs, 10X High Fidelity PCR Buffer, 50 mM MgSO4, and 5 µM each of forward and reverse primers (Additional file 1: Table S2) and mixed thoroughly before adding 2.5 U Platinum® Taq High Fidelity. PCR reactions used 250 ng genomic DNA in a total volume of 50 μl. An ABI 9600 thermal cycle was used to run the long-range PCR program: 94°C for 2 min; 15 cycles at 94°C for 15 sec, 65°C for 30 sec, 68°C for 20 min; 20 cycles at 94°C for 15 sec, 65°C for 30 sec, 68°C for 20 min 20 sec; and 68°C for 7 min.

Fosmids and long-range PCR products were sequenced using shotgun-sequencing protocols with dye-terminator sequencing as outlined previously [9,25]. Sequences have been deposited in GenBank with the following accession numbers: JX008026 (HIP01829), JX008027 (HIP05435), JX008028 (HIP08327), JX008029 (HIP09410), JX008030 (HIP09453), JX008031 (HIP09498), JX008032 (2DS2 deletion), and JX008033 (3DL2-Fcar). To detect the 3DL1/L2 hybrid gene, we sequenced exons (exon3-5 and exon7-9) using gene specific sequencing primers (Additional file 1: Table S1) on 3DL1/L2 hybrid long-range amplicons. For sequence analysis, we used full genomic sequence of the 3DL1/L2 hybrid from EU267269. Alleles and haplotypes have been named according to the guidelines established by the KIR Nomenclature Committee [26] and deposited into IPD-KIR (http://www.ebi.ac.uk/ipd/kir/).

### Computational analyses

#### Gene annotation

The finished genomic sequences were analyzed using cross-match of the Phred-Phrap-Consed package [27,28], which was also used extensively for annotation. Annotation of the sequences used updated KIR cDNA and genomic sequences from the IPD-KIR database (http://www.ebi.ac.uk/ipd/kir/).

#### Sequence analysis

Sequences of the individual genes were aligned and phylogenetic analysis was performed using the FSA multiple alignment server [29] or MAFFT [30] and manually corrected in BIOEDIT (http://www.mbio.ncsu.edu/BioEdit/bioedit.html). Alignments were viewed and analyzed on Geneious v5.5 (http://www.geneious.com). Recombination analysis was performed by using Recombination Detection Programs (RDP) version 3 [31] with a sliding window of 20 bp, with Bonferroni correction and a threshold of P = 0.001. The neighbor joining (NJ) analysis was performed using MEGA version 5 (http://www.megasoftware.net/) [32]) with 1000 replicates, pairwise deletion, midpoint rooting, and the Tamura-Nei method. The repeat sequence in the breakpoint was identified by using censor server [33].

#### Analysis of KIR haplotype structures

KIR haplotypes were determined based on the previously sequenced KIR haplotypes [6,9,34] and the copy number of each KIR gene in an individual. The linkage disequilibrium (LD) among the genes and the frequencies of KIR haplotypes were calculated by the maximum likelihood method using Arlequin v3.5 [35]. Primer sequences used in generating the haplotyping amplicons and are listed in Additional file 1: Table S3.

## Results

**Figure 1:**
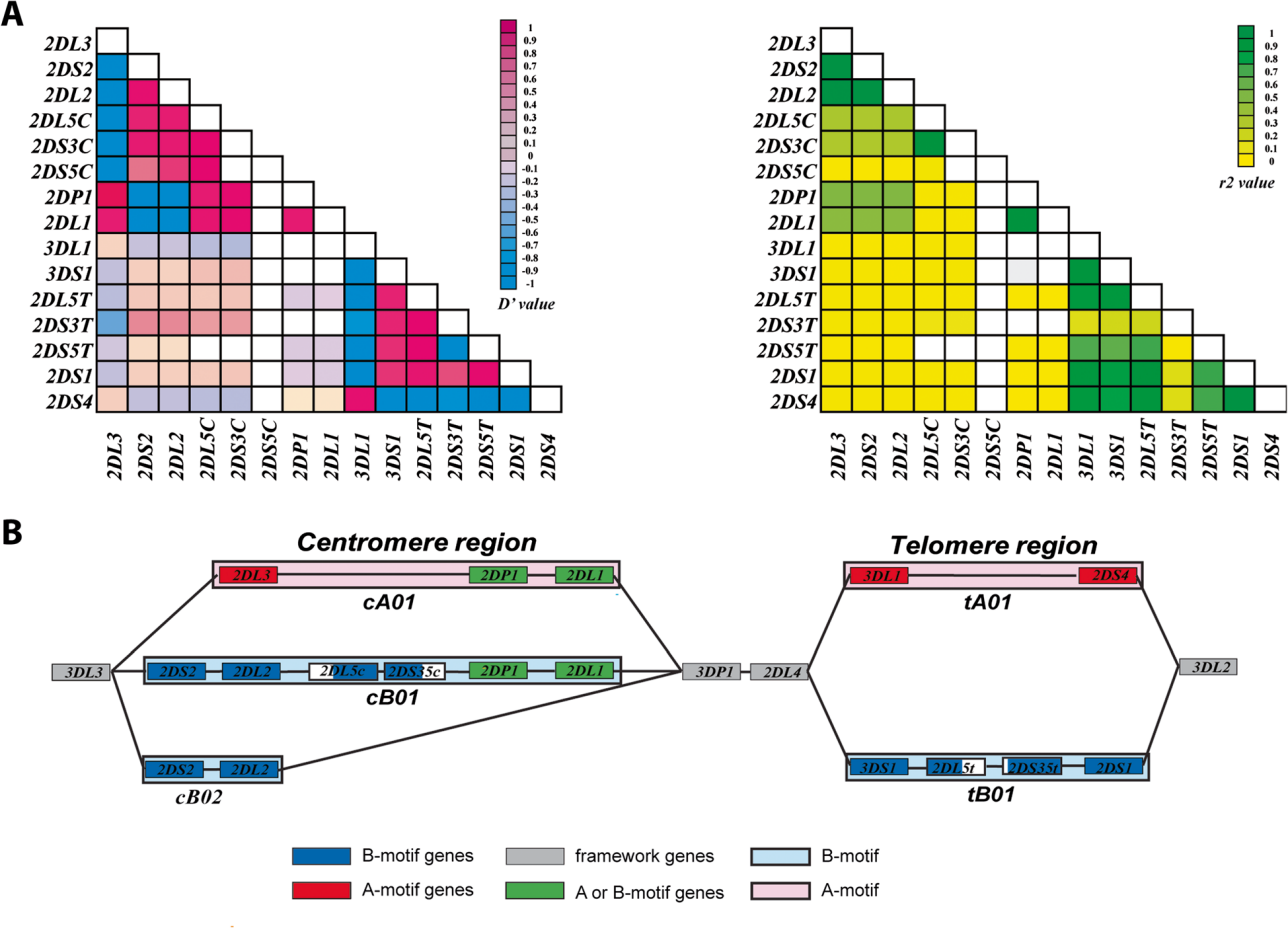
**Linkage Disequilibrium among KIR genes.** (**A**) Two LD maps of the KIR region were constructed using Arlequin v3.5, comparing D’ and r^2^ values between pairwise combinations of the 16 KIR genes. Each block on the maps represent either the D’ (left map) or r^2^ (right map) value calculated. The two color keys indicate the range of values represented by different colors. Gene names are shown at the left and bottom of each map. (**B**) A cartoon representation of the KIR region, showing the combinations or blocks of KIR genes into commonly seen motifs. The color-coded key at the bottom indicates the motif or region that each gene is linked to, with the 2DL5-2DS35 block hatched to highlight both centromeric and telomeric residence.

Complete genomic sequences of the major KIR haplotypes enabled the design of an approach to detect KIR gene-content haplotypes. Our primary design was based on linkage disequilibrium (LD) derived from 27 completely sequenced haplotypes and a diversity panel of 384 DNAs using STSs registering presence or absence of loci as previously described [9]. We calculated D’ and r^2^ between each of the haplotype-variable *KIRs* and mapped the coordinates among all pairwise combinations (Figure 1). These calculations were used to direct our design of methodology to detect known haplotypes and to uncover new arrangements that could be resolved by further genomic sequencing.

### KIR gene content haplotyping – methods development

Gene content haplotype mapping was carried out using PCR sequencing of amplicons, each designed to detect multiple *KIR* loci. These assays were initially based on the 27 published complete genomic haplotype sequences and used to test samples from a diversity panel consisting of 96 each of Asian, African, Caucasian, and Hispanic samples. Further development in methodology occurred over iterative applications. DNAs yielding results that did not correlate with any known haplotype structures were subjected to genomic sequence analysis to obtain detail on novel structures and assays were modified to incorporate new data obtained. Fourteen assays were eventually developed that met the following criteria: *(i)* each assay produced a PCR product regardless of the haplotype combination being queried, thus removing PCR failure as a source of false negative results; *(ii)* PCR sequencing of the collective products provided at least 2 independent confirmations of the presence of any particular locus; *(iii)* quantitative measurement of relative peak heights was available to predict copy number variation of related loci; and *(iv)* the sum of the results could be used – given a haplotype structure rule set – to predict unambiguously the diploid haplotypes within any individual examined.

Testing of the initial amplicon designs on a diversity panel of DNAs showed that in most cases results were consistent with expectations based on the known haplotype sequences. In assigning genes to specific haplotypes, the following assumptions were made, based in part on calculated LD values (relevant D’ and r^2^ are taken from Figure 1): *(i)* The order and position of each *KIR* gene is invariant; *(ii)* The 2DL5-2DS35 gene block is located on either the centromere or telomere based on the presence and copy number of adjacent *KIR* genes (centromere-2DP1 and 2DL1; telomere-3DS1-2DS1); *(iii)* Some genes are completely segregated to discreet haplotypes (*e.g.* 2DL3 versus 2DS2-2DL2). If an individual has both 2DL3 (or 2DS1) and 2DS2-2DL2 (or 3DL1-2DS4) from the gene content measurement, each was assigned to a different haplotype; *(iv)* Blocks with strong LD (*e.g.* 2DS2-2DL2/2DP1-2DL1/3DP1-2DL4/3DL1-2DS4/3DS1-2DL5-2DS35-2DS1) were used as a secondary check of haplotype assignments; *(v)* The 2DP1-2DL1 block is connected with either 2DL3 or 2DL5-2DS35 (no case of a 2DS2-2DL2 connection was found); *(vi)* 3DP1-2DL4-3DLS1/3DP1-2DL4-3DS1-2DL5-2DS35 can be duplicated (−ins3, -ins4, -ins5) or deleted (−del5, -del6, -del7) in a haplotype.

DNAs that did not yield patterns consistent with these criteria were further analyzed using long-range PCR where the suspected regions could be amplified conveniently and fosmid cloning where the regions were larger or otherwise complex. Once new sequence data was acquired, the KIR genotyping amplicons were redesigned to incorporate the new genomic structures. This approach allowed our designs to converge on an optimal set of amplicon sequences and specific variant positions that could detect all *KIR* loci (Table 1). A somewhat more vexing issue arose when attempting to determine gene copy number due to limitations in our design and dye-terminator sequencing. This issue was resolved satisfactorily by comparing RT-PCR analysis of samples to unambiguously determined gene copy number and establish the identity of cell lines containing previously undetected duplications or deletions. After determining the genomic sequence of variant motifs, we were able to focus the design of amplicons using variant dye terminator peak heights at specific positions to determine gene copy numbers (Additional file 2: Figure S6). These data provided additional confirmation of gene presence (Table 2).

### KIR gene content haplotypes

**Figure 2:**
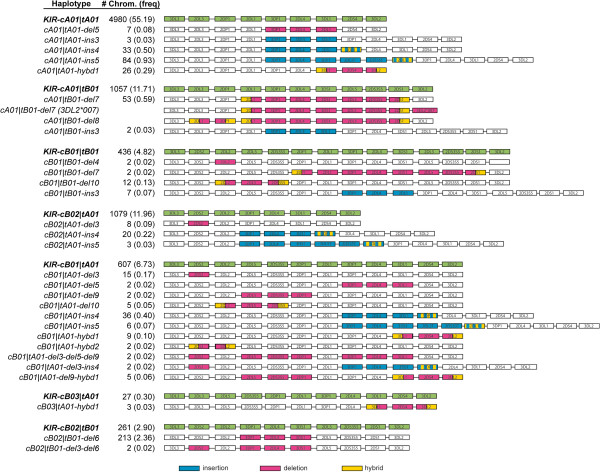
**Extensive KIR haplotype diversity found in extended populations.** The results of KIR haplotype determination on 4,512 individuals are summarized. The names of the KIR haplotypes are listed in the left-hand column, were the previously sequenced haplotypes are in bold and corresponding recombinant haplotypes immediately beneath. Nomenclature for the recombinant haplotypes identified in this study is based on the presence of an insertion (ins), deletion (del), or gene hybridization (hybd) of a segment of the parent haplotype. The number of chromosomes, frequency of occurrence (the cA01|tB01-del7 and del8 results were not separated), and a cartoon depicting the gene content structure of each haplotype are indicated immediately to the right. A color-coding key at the bottom indicates the gene rearrangement types found within each recombinant haplotype.

From a total of 4,512 DNAs genotyped – combined with genomic sequencing where needed – we were able to unambiguously define a total of 37 KIR haplotypes (Figure 2). We also detected 10 additional structures that were distinct but only present in a single copy in this sample set. It was possible to predict haplotype structures for these samples but we did not confirm them through sequencing and thus do not report them here. The nomenclature for KIR haplotypes is based on the division of the region into centromeric and telomeric patterns [36]. Using this division as a guide, it was possible to summarize the altered patterns – those found in this study and previously – into 10 types of alterations including some previously described motifs (Table 3). We chose to include the cB03 motif in the submotif category since it is rare and can be described as an insertion of the 2DL5-2DS35 block into the cA01 motif. Together the submotifs include single and multiple gene deletions and insertions and hybridizations of genes associated with specific gene expansions. Many can be found in multiple associated haplotypes, although often within a single major motif (*e.g.* hybd1 and tA01). Overall the frequency of any one of these mutant patterns is low and collectively account for about 7% of the total haplotypes detected in our study.

**Table 3:** Summary of KIR haplotype motif frequencies

**Major patterns**			
**Centromere**		**Number**	**Freq (%)**
*cA01*		6036	66.93
*cB01*		1042	11.55
*cB02*		1340	14.86
**Telomere**			
*tA01*		6728	74.61
*tB01*		1770	19.63
**Submotif patterns**	**Associated region**	**Number**	**Freq (%)**
***cB03***-2DL5-2DS35 insertion	cA01	22	0.24
***del3***–2DS2-deletion	cB01, cB02	24	0.27
***del4***-2DL2-deletion	cB01	2	0.02
***del9***–2DL5-2DS35-2DP1 deletion	cB01	2	0.02
***del10***–2DL2-2DL5-2DS35 deletion	cB01	17	0.19
***hybd1***–3DL1-3DL2 hybrid/2DS4 deletion	tA01	35	0.39
***del5***–3DP1-2DL4-3DL1 deletion	(cA01/cB01)|tA01	10	0.11
***del6***–3DP1-2DL4-3DS1 deletion	(cA01/cB02)|tB01	214	2.37
***del7 or del8***–2DP1(partial)-3DP1-2DL4-3DS1-2DL5-2DS35-2DS1(partial) deletion	(cA01/cB01)|tB01	55	0.61
***ins3***–3DP1-2DL4-3DL1 insertion	(cA01/cB01/cB02)|tA01 (cA01/cB01)|tB01	14	0.16
***ins4***–3DP1-2DL4-3DS1 insertion	(cA01/cB01/cB02)|tA01 (cA01/cB01)|tB01	91	1.01
***ins5***–3DP1-2DL4-3DS1-2DL5-2DS35-2DS1(partial) insertion and additional recombination with 2DP1	(cA01/cB01/cB02/cB03)|tA01 cB01|tB01	95	1.05
**Combined submotifs**	cB01|tA01 cB02|tB01	14	0.16
**Singlets**		10	0.11

### Deletion mutants in the cB01 motif

**Figure 3:**
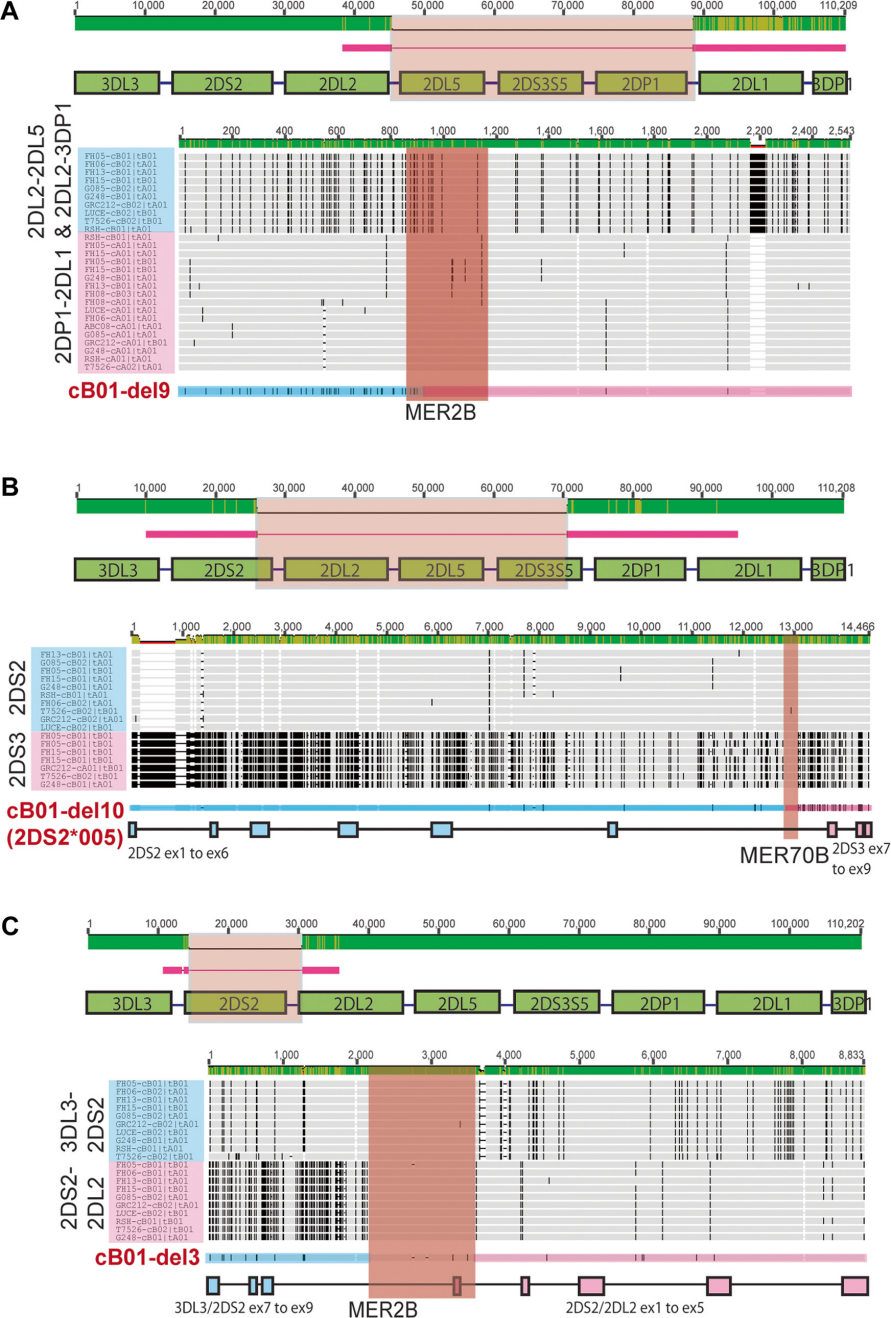
**Gene deletions within the cB01 motif may have involved MER repeat elements.** (**A**) The cB01-del9 structure derived from DNA source HIP09410M, (**B**) The cB01-del10 structure derived from DNA source HIP05435M, and (**C**) The cB01-del3 structure derived from DNA source IHW30048M. For all, sequences were compared with a reference cB01 motif consensus sequence compiled from 6 previously sequenced haplotypes [9]. Red lines represent the extent and portions of the mutant haplotypes that were sequenced relative to the cB01 motif, with the sequences absent represented with thin lines inserted for alignment. Immediately beneath each cartoon, each mutant haplotype sequence was aligned against two groups of wild-type sequences (outlined in blue and pink) encompassing the ends of the predicted deletion point in each mutant. The genes or intergene regions being aligned are listed vertically at the left and the derivative cell lines and corresponding haplotype names are listed within the blue or pink shading. The patterns of mismatches (black hatches) indicate the positions of minor allele SNPs among the aligned sequences. Immediately beneath, the mutant haplotype is aligned with corresponding SNP markings and blue and pink shading indicating the sequence origin, above a similarly color-coded cartoon depicting the gene structure where appropriate. The positions of repeat elements within each pair of regions flanking the deletion are indicated with the aligned sequences highlighted in orange shading above the name of the repeat element. In the two scales, the upper for the genomic region and the lower for the gene sequences, the lighter shading in the green bar indicates the extent of sequence divergence among the aligned sequences. Supporting phylogenetic and breakpoint analyses are presented in supplementary Additional file 2: Figure S2.

The high homology among *KIR* genes and the tandem gene structure of the region suggested possible recombination events that may have occurred to generate each pattern, some of which may later have recombined into other motifs in further expansions. In the centromeric cB01 motif, three different deletion mutants were found: one deletion removed a single locus (del3) and two others removed regions encompassing 3 loci (del9 and del10). In order to gain insight into possible mechanisms leading to these deletions, we mapped the deletion endpoints by comparing examples of each of the recombinant sequences incorporating the deletion with previously determined genomic sequences. In each case it was possible to identify a probable deletion boundary that overlapped with a repetitive genomic element found duplicated at the boundaries of the parent genomic structure (Figure 3). Two distinct MER transposable element repeats [37] were found, with MER2B found in common between the del3 and del9 structures. The MER2B elements encompassing the del9 block of genes within the cB01 parent motif indicate a relatively precise crossover point within the 309 bp of homology between the flanking repeats because of the 5% sequence divergence in the 5’ half of the repeats. Conversely, the precise recombination point is arbitrary within the gene inter region (over 1100 bp) in both 3DL3-2DS2 and 2DS2-2DL2 because of the 99% sequence homology between groups of transposable elements (MER2B-AluSg-MER2-L1ME_ORF2-AluS) in the parent cB01 motif. The deletion that led to the del10 submotif of cB01 and the 2DS2/S3 hybrid gene (2DS2*005) [38,39] appears to be focused on a 357 bp MER70B repeat shared between the 2DS2 and 2DS3 intron 6 sequences (98% sequence homology) flanked by what is otherwise relatively highly divergent sequence. As a general statement, perhaps not surprisingly, it appears that repeat elements not only have been active in shaping the *KIR* region over long spans of primate evolution [40], but also continue to do so in more recent times within the human population.

### Recombination in the telomeric region generating hybrid genes

**Figure 4:**
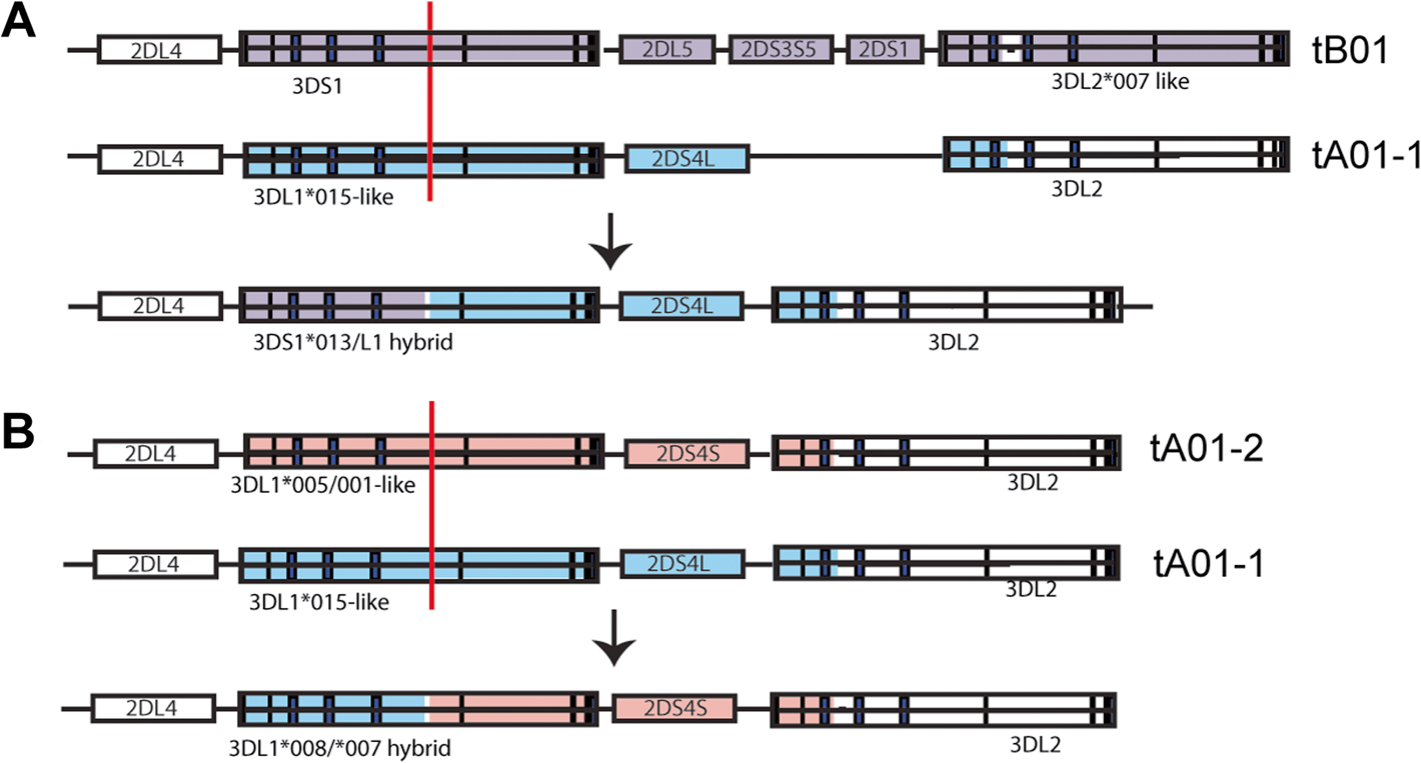
**Linkage disequilibrium and hybrid gene exchange in the telomeric KIR region.** (**A**) Depicted are two major 3DL1/S1 motifs in the telomeric region and their linked genomic sequences indicated with color shading. Supporting phylogenetic analysis of the represented linkage disequilibrium arrangements were found from an examination of all sequenced KIR haplotypes (Additional file 2: Figure S2B). Exchange between 3DL1 and 3DS1 connecting 3DS1-intron 5B in tB01 with 3DL1-intron 5B in tA01-1 leads to the generation of hybrid alleles of telomeric 3D genes, as depicted. (**B**) Essentially similar depiction of 3DL1*007/*008, connecting 3DL1-intron 5B in tA01-1 with 3DL1-intron 5B in tA01-2. Color shading indicates origin of the sequences. For both (**A**) and (**B**) red bars within gene structure cartoons represent possible recombination sites in the generation of hybrid genes. Supporting phylogenetic, alignment, and breakpoint analyses are presented in Additional file 2: Figures. S2B and S3.

One of the unique features of the 3DL1/S1 locus is linkage disequilibrium (LD) of adjacent regions with specific allelic variation at 3DL1/S1. Immediately adjacent to 3DL1/S1 is the 2DS4 locus, which can be divided into two functionally different allele groups 2DS4L and 2DS4S. These groups, each of which contains several alleles, are distinguished by a 22 bp deletion in exon 5 of the 2DS4S group relative to the 2DS4L group. This deletion introduces a frameshift, predicting a truncated KIR2DS4 protein that may be secreted due to loss of the transmembrane and cytoplasmic domains [41]. Phylogenetic analysis of 29 haplotype sequences described here and in our previous report demonstrated strong LD between the 3DL1*015-like alleles and the adjacent 2DS4L group of alleles. Similarly, the 3DL1*005/001-like group appears uniquely associated with the 2DS4S group and the 3DS1 group uniquely linked to the 2DL5-2DS35-2DS1 block of genes (Figure 4 and Additional file 2: Figure S2C). Both associations were recently reported by Hou *et al.*[42]. This division is noteworthy because it is likely that these groups may comprise three ancient lineages of inhibitory and activating receptors encoded by this locus [43].

Using this LD relationship among the haplotypes, it was possible to predict the putative origins of the 3DL1/S1 hybrid genes described in this report and in previous studies. Sequence alignments pointed to a putative exchange segment in intron 5 flanked by repetitive elements in common with both recombinant structures. Pairwise identity plots more precisely pinpointed the recombination events that could have resulted in the 3DS1*013/L1 and 3DL1/L2 (3DL1*059) hybrid genes (Figure 4B and Additional file 2: Figure S3) [44]. These data may emphasize the importance of considering highly local linkage disequilibrium within *KIR* in genetic association studies because an association with a *KIR* locus or allele may indicate causation, linkage, or causation through epistatic interactions between linked loci.

### A KIR3DL2 deletion event

**Figure 5:**
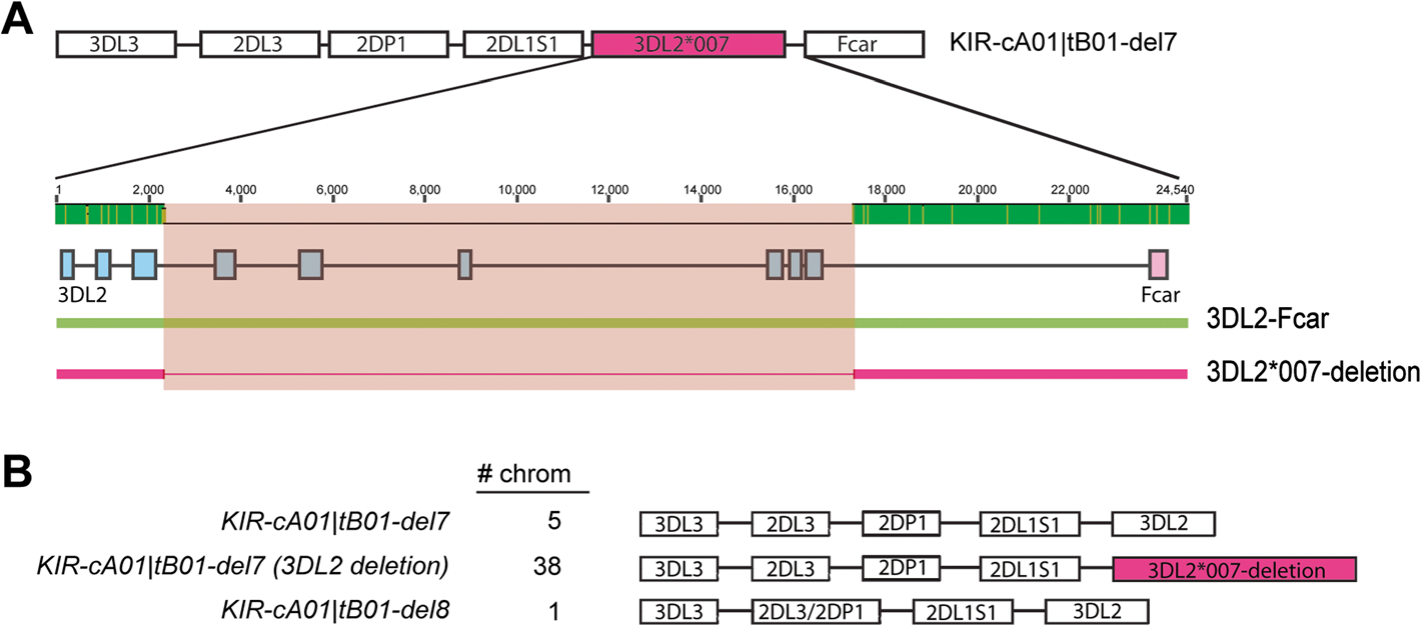
**The KIR cA01|tB01-del7 haplotype has alternate forms, with and without a KIR3DL2 partial gene deletion.** (**A**) A cartoon depicting the cA01|tB01-del7 haplotype and, expanded beneath, the structure of a partial deletion of 3DL2 (from exon4 to exon9) found in a major subset of the cA01|tB01-del7 mutant haplotypes examined. Based on a sequence alignment of wild-type 3DL2 (green line) and mutant 3DL2*007 (red line with thinning indicating absent sequence), the deleted region spans ~14 kb from intron 3 to the 3’UTR flanked by repeat elements at both breakpoint sites. The 3DL2 and Fcar exon structures are indicated by blue and pink boxes, respectively. Pink shading overlays the region deleted. The lighter shading within the green bar at the scale indicates the extent of sequence divergence between the aligned sequences. (**B**) Three distinct derivative haplotypes were found in the populations studied. The number of chromosomes detected for each haplotype is indicated adjacent to the haplotype name and a cartoon depicting the structure. All samples were examined by long range PCR-sequencing with the deletion found only in individuals with the 3DL2*007-like allele (based on exons 1–3) and the –del7 structure.

Previous examination of *KIR* genomic sequences described the contraction of *KIR*, generating two haplotypes with 4 and 5 *KIR* genes each [34]. Our studies had also identified these haplotypes using the KIR haplotyping approach described here, but in most cases found a discrepancy in the number of *KIR3DL2* present, usually detecting 1 copy per individual versus the expected 2 copies. Upon further examination of genomic sequences from the discrepant individuals, we found that most of the cA01|tB01-del7 haplotypes in our study in fact contained a deletion of *KIR3DL2* from intron 3 to beyond exon 9 that spanned a region of about 15,000 bp (Figure 5). The deleted region included the portions of *KIR3DL2* that were used as part of our gene copy determination (Table 2), thus explaining the discrepant genotyping result. When we expanded our analysis to include all individuals in our study, we found the frequency of the KIR3DL2 deletion included 38 of the 42 cA01|tB01-del7 haplotypes detected. In addition, all of the deletion haplotypes contained the exons 1 to 3 sequences of the KIR3DL2*007 allele, suggesting a recent common origin. Essentially similar long-range genomic sequencing showed that the two cA01|tB01-del8 haplotypes detected in our panel did not contain this deletion. A recent study showed a similar deletion pattern on the same haplotype structures [45].

### Unequal recombination and new telomeric motifs

**Figure 6:**
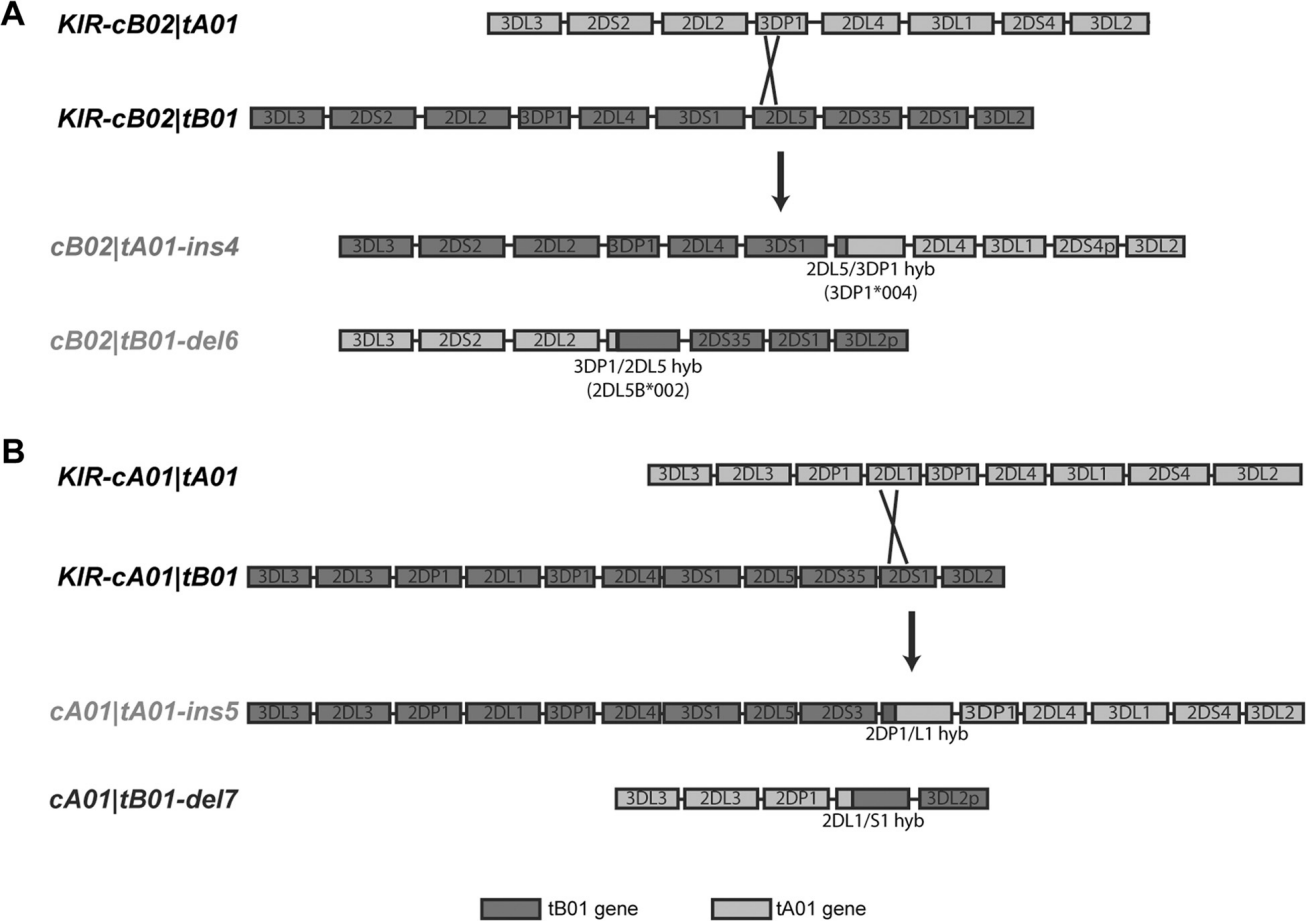
**Putative patterns of exchange in the generation of mutant arrangements of combined motifs in the telomeric region.** (**A**) Exchange between the KIR-cB02|tA01 and KIR-cB02|tB01 haplotypes yields the KIR-cB02|tA01-ins4 and the KIR-cB02|tB01-del6 haplotypes, gaining and losing respectively the 3DP1-2DL4-3DS1 gene block. (**B**) Exchange between parent haplotypes KIR-cA01|tA01 and KIR-cA01|tB01 yields two KIR mutant haplotypes cA01|tA01-ins5 and cA01|tB01-del7, gaining and losing respectively the 2DL1-partial-3DP1-2DL4-3DS1-2DL5-2DS3-2DS1-partial gene block. The names of haplotypes sequenced in this report are in light gray. The sequence of the cA01|tB01-del7 haplotype was described in a previous report [34]. In both A and B, cartoons depicting the haplotype motif structures are color-coded according to the key at the bottom. Crossed lines indicate putative recombination points with detailed supporting breakpoint and alignment analysis presented in Additional file 2: Figure S4.

In the telomeric region, it was possible to identify a number of rearrangements, including duplications, deletions, and gene hybridizations. The gene expansion represented by the ins4 expansion, where *KIR3DP1, KIR2DL4*, *KIR3DS1*, and a portion of *KIR2DL5* are inserted into the tA01 region, is found in recombinants of four of the major haplotypes and results in one of two examples of *KIR3DL1* and *KIR3DS1* in phase on a single chromosome. This region and surrounding gene regions were sequenced from a single individual and data were incorporated into the genotyping strategy to enable identification of the duplicates of *KIR3DP1* and *KIR2DL4* and multiple *KIR3DLS1* marking the expanded segment. Subsequent genotyping detected this expansion combined with 4 of the 5 distinct centromeric KIR haplotype patterns. Alignment of the cB01|tA01 and cB02|tB01 haplotypes points to two possible recombination points between these haplotypes that could have generated the tA01-ins4 expansion and would also yield the reciprocal tB01-del6 deletion found in this study (Figure 6A). This pattern of insertion and deletion was also characterized by population and family studies [46-49]. Sequence alignment and breakpoint analysis indicated that the putative recombination event may have taken place between *KIR2DL5* and *KIR3DP1* and localized to the intron 2 regions in the parent haplotypes (Additional file 2: Figure S4).

A similar misaligned recombination event can be postulated to have given rise to a second expansion in the tA01 segment, duplicating the 3DP1-2DL4-3DS1-2DL5 gene block and portions of the flanking 2DL1 and 2DS1 genes to yield the ins5 mutant structure (Figure 6B). This structure is found in combination with three different centromeric *KIR* segments and represents the second example of duplication of *KIR3DLS1* with *KIR3DL1* and *KIR3DS1* separated in phase. The reciprocal recombinant from this postulated event would yield the cA01|tB01-del7 haplotype – missing the same gene block – previously described [34]. A caveat for this postulated recombination event is the resultant 2DP1/L1 hybrid gene found in the ins5 structure, which is not reflected in the reciprocal del7 structure where the 2DL1/S1 hybrid is found. Sequence alignments and breakpoint analysis lend strong support to these hybrid structures (Additional file 2: Figure S4). A complete sequence of the cA01|tA01-ins5 haplotype with overlapping fosmids covering the hybrid gene was obtained (Additional file 2: Figure S1), most likely ruling out a sequence artifact. Therefore, in order to accommodate the recombination event modeled, a secondary gene conversion event between a 2DS1/L1 hybrid in the original tA01-ins5 chromosome and the 2DP1 exons 1–3 region is postulated.

### Migration of the 2DL5-2DS35 gene block

One of the interesting substructures of the KIR locus includes the 2DL5-2DS35 gene block, found in both the cB01, cB03, and tB01 regions, with homology between the centromeric and telomeric versions at over 98.7% over the extended 26 kb region [50,51]. Based on the genomic sequences from this and our previous study [9], we constructed a putative series of two successive unequal recombination events that could have moved the centromeric 2DL5-2DS35 gene block to the telomeric region (or vise versa). Phylogenetic analysis of the 2DL5 exons 1–2 genomic region showed clear differentiation of 2DL5A and 2DL5B alleles. Based on fully sequenced KIR haplotypes, the 2DL5A group is located on the telomere region and the 2DL5B group is located on the centromere region (Additional file 2: Figure S5). Phylogenetic analysis of the intron3 to exon9 region showed that some 2DL5 alleles (2DL5A*00501 and 2DL5B*00201) associated with the 2DS3 gene while other alleles (2DL5A*001, B*00601, B*00801 and B*00401) associated with the 2DS5 gene. Thus, the exon1 to exon2 region might indicate the location of the 2DL5-2DS35 gene block (centromere or telomere) while the downstream region (intron3 to exon9) might be predictive of linkage association (2DS3 or 2DS5).

**Figure 7:**
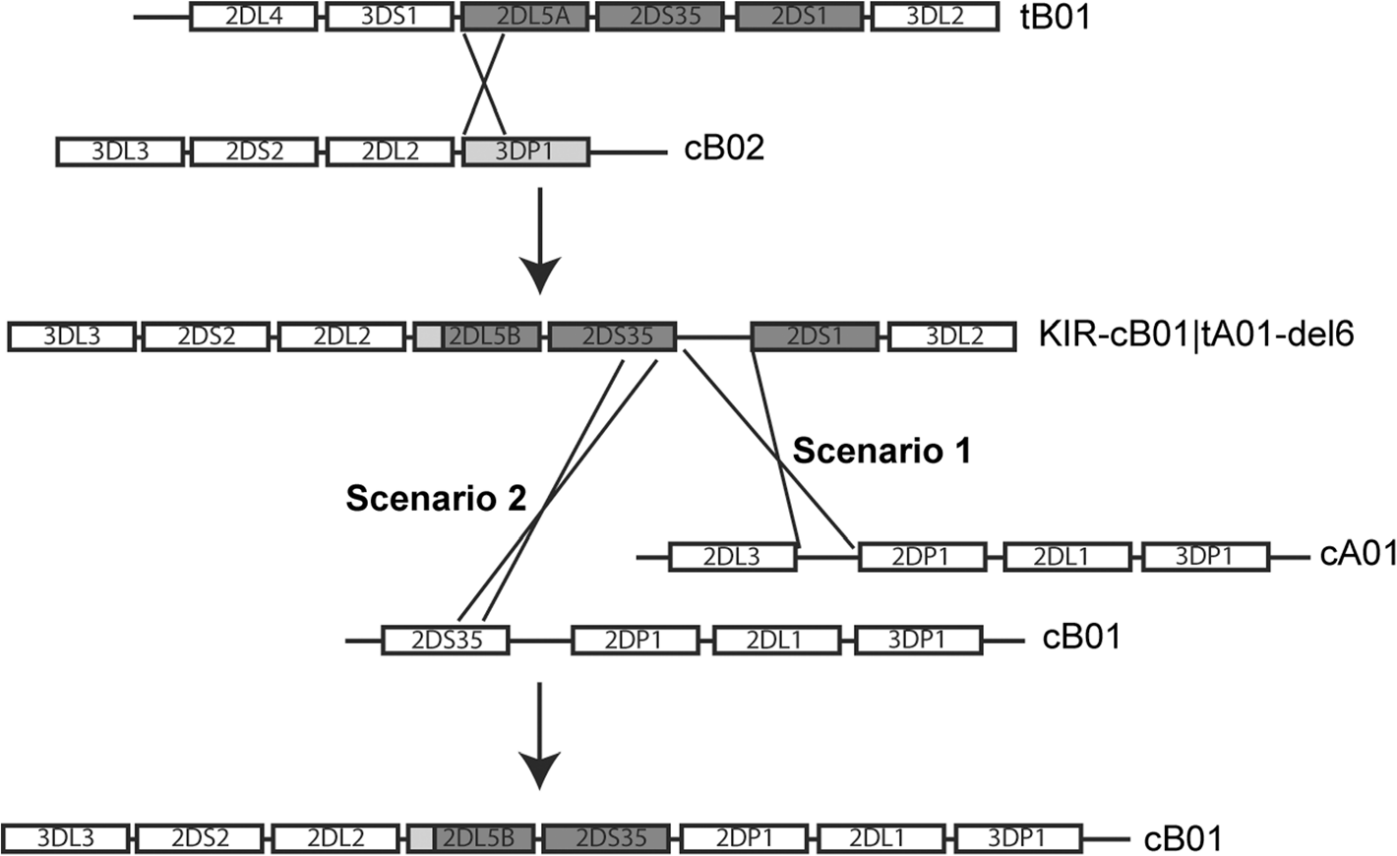
**Putative recombination pathway for transfer of the 2DL5-2DS35 gene block between the centromeric and telomeric motifs.** An exchange between the intron 2B regions of 2DL5A (tB01) and 3DP1 (cB02) is depicted, yielding the KIR-cB02|tB01-del6 haplotype. A second exchange between the intergenic regions of 2DS35-2DS1 in KIR-cB02|tB01-del6 and 2DL3/2DS3/S5-2DP1 in the cA01 or cB01 motifs is depicted yielding the cB01 motif with resultant transfer of the 2DL5-2DS35 gene block. Supporting alignment, breakpoint, and phylogenetic analyses for this pathway are presented in Additional file 2: Figure S5.

Sequence alignments and breakpoint analysis of the 2DL5-2DS35 gene block indicated that the first step in this transfer may have been mediated by a recombination event between the intron 2B regions of 2DL5A in the telomeric segment and 3DP1 from the centromeric region (Figure 7). This event connects 2DL5A-2DS35-2DS1 in the tB01 motif to 2DL2 in the cB02 motif, resulting in the KIR-cB02|tB01-del6 haplotype in which 3DP1-2DL4-3DS1 is deleted (step 1 in Figure 7). This deletion was previously reported in Norman *et al.* and Gomez-Lozano *et al.*[48,49]. As part of this event, sequence exchange of the exon1-exon2 region between 3DP1 and 2DL5A would convert the 2DL5A allele into the 2DL5B allele, which is specific to the centromeric region (Additional file 2: Figure S5). For the second step, the KIR-cB02|tB01-del6 haplotype is postulated to undergo a recombination event with the cA01 motif in the inter-gene regions of 2DS35-2DS1 and 2DL3-2DP1 or with the cB01 motif in the intron6 region of 2DS35 to generate a new cB01 motif [9]. Perhaps the appealing features of this model are its relative simplicity – two steps – and that all of the structures in the pathway are presently found in populations.

## Discussion

The human *KIR* locus, with its unique repetitive structure has facilitated numerous asymmetric recombinations that duplicated *KIR* genes, deleted *KIR* genes, and formed new hybrid *KIR* genes with novel ligand-binding and signaling functions [52]. A unique sequence in the *KIR* locus is located in the 14 kb intergenic region that separates *KIR3DP1* from *KIR2DL4* and divides the locus into centromeric and telomeric segments of similar size [7,9]. This unique sequence has been the site for events of reciprocal recombination to form new variant *KIR* haplotypes [6,53]. Recombination at this site is evidently a major driver of *KIR* haplotype variation as it accounts for most of the major haplotypes, which involve combinations of the three common centromeric and two telomeric motifs [9]. A minor percentage of recombinant haplotypes, including those newly reported in this study, may have arisen from other mechanisms of recombination including insertions and deletions of gene blocks that may have involved misalignment at repetitive sequences in common between different *KIRs*. The resources produced from this study include two new complete haplotype sequences and 6 partial sequences of duplicated or deleted regions representing submotifs found in one or more haplotypes.

The high mutability of the *KIR* region is likely driven by immunological processes, which contribute a central role in the human immune response for KIR receptors, including infection and reproduction [54]. In line with this, *KIR* genetics have been widely studied in relation to complex disease [10]. However, the genotyping methods used in most of those studies measured the presence or absence of specific *KIR* genes with reduced or absent ability to reference haplotype structures. Knowledge of the complete haplotype structure is likely important when attempting to identify causative *KIR* genetic factors associated with disease for a number of reasons, including LD and possible epistatic interactions among *KIR*. Given the complexity of *KIR* allelic polymorphism combined with gene content haplotype polymorphism, perhaps the optimal data set is the complete genomic sequences of each pair of haplotypes in an individual (*i.e.* all of the genetic variation present). However, until technology allows for such data to be derived economically, methods that determine haplotype structures, supplemented with allelic information, mark progress towards the identification of *KIR* genetic factors that are directly involved or causative of complex diseases.

The *KIR* haplotyping method described for this study is currently based on an ABI dye-terminator sequence-based approach that detects specific variant positions identifying *KIR* genes and ratios at SNP positions determining gene copy number. Our reliance on sequence-based methods stems from the desire to use uniform methodology in genotyping in our laboratory. However, this approach can be adapted to other sequencing methods that detect specific SNPs and allow for quantitative determinations at SNP positions. Regardless of the genotyping approach, robust information processing of the data is needed, especially when scaling studies into the thousands of samples. Again, although we developed in house software for this purpose, appropriate algorithms can be developed within a variety of different software frameworks. The overall cost of the assay is similar to existing *KIR* gene presence/absence methods while yielding potentially valuable more complete genetic information, including phase and copy number.

Of the 9,024 chromosomes examined, 10 samples showed unique genotyping patterns, each different from one another and from the 37 haplotypes described. It was not possible to assign a unique structure to these haplotypes using the existing set of insertion, deletion, and hybridization submotifs. In addition, we did not pursue proving these structures through sequencing due to their single occurrence. There are likely other rare recombinant types not yet described with frequencies in the very rare range, perhaps similar to the frequency of new HLA alleles still being discovered. In addition, our survey populations did not include substantial numbers of African, Asian or Hispanic individuals (in the hundreds each), leaving much of the frequencies to be determined for those populations and with numerous new haplotypes likely to be found in the more diverse African populations. Excluding these 10 very rare haploytpes, the sequence-validated rare haplotypes – those with submotif patterns (Table 3) – collectively account for almost 7% of the total we examined here. These numbers may be significant in studies when the copy number or tandem arrangement of genes is important [55]. In addition, when combined with allelic variants that significantly alter expression of KIR genes [56] the exceptional cases can collectively amount to significant percentages. This may prove to be an important perspective for *KIR* and complex disease, especially in light of studies where rare variants collectively may be causative of complex diseases [57].

Among the rare mutations that may deserve unique attention are those that delete the framework genes *KIR3DL3*, *KIR2DL4*, and *KIR3DL2*, which may perform essential functions given their near invariant presence in all KIR haplotypes compared with more variable presence of other *KIR* within a haplotype. In this study, while no cases of a *KIR3DL3* deletion were observed, the *KIR2DL4* deletion found in the cB02|tB01-del6 haplotype accounted for about 2.3% of the haplotypes surveyed. The KIR2DL4 protein has been implicated as the receptor for the HLA-G ligand and may function in the pregnant environment [58] although it may not play an essential role there as maternal homozygous deletion variants can apparently achieve a successful pregnancy [59]. The KIR3DL2 protein has been shown to interact with specific HLA-A ligands and may act as an inhibitory receptor [60,61]. As a framework *KIR* locus, *KIR3DL2* shares with *KIR2DL4* a rare variant type. The cA01|cB01-del7 haplotype is the only haplotype found where both genes are deleted. Functional studies performed on lymphocytes derived from individuals with this haplotype in a homozygous or hemizygous state could be revealing.

No doubt additional functional data about individual KIR receptors and their ligands are needed before conclusive causative genetic associations of *KIR* can be made with complex diseases. However, as new functional understandings are revealed, and as *KIR* population genetics is more comprehensively defined and genotyping becomes more extensive, refined, and economical, we can fully expect to achieve this long sought goal.

During the course of submitting this paper, a parallel study on KIR haplotypes was published where 72 haplotype structures were reported [45]. Three differences between our reporting of haplotypes and that study account for the numerical difference. First, in our descriptions we did not consider haplotypes that carried the 2DS3 or 2DS5 genes or the 2DS4L and 2DS4S groups as distinguishing and instead considered the 2DS3/5 genes and the 2DS4L/S allele groups as sets of alleles. Also, among the 72 reported, 31 were single occurrence, none validated by sequencing, and none of which intersected with haplotypes from our samples. Although we detected 10 haplotypes for which we could estimate structures in addition to the 37 we reported in Figure 2, each had only a single occurrence in our population set, and we chose not to fully characterize them through sequence analysis. Our standard for reporting new haplotypes was determination by our genotyping assay and sequencing of genomic DNA (fosmids or LR-PCR) when new structures were uncovered.

## Conclusions

Knowledge of the complete haplotype structure of KIR is critical for association studies between KIR genetic variation and complex diseases. We developed a method to detect phased KIR gene-content haplotypes based on PCR sequencing of amplicons. Using this method, we defined a total of 37 KIR haplotypes from genotyping 9,024 chromosomes. An additional 10 haplotypes were detected in single copy but were not confirmed by sequencing. The 37 KIR haplotypes were sorted into 10 types of structural alterations, including gene deletions, insertions, and hybridizations, which together suggest a number of recombination events that might have occurred during KIR evolution. This haplotyping method is an important step towards the identification of KIR genetic factors that are associated with complex diseases.

## Abbreviations

HCT: Hemapoietic Cell Transplantation; KIR: Killer cell Immunoglobulin-like Receptor; LD: Linkage Disequilibrium; MHC: Major Histocompatability Complex; NK: Natural killer cell.

## Competing interests

The authors declare that they have no competing interests.

## Authors’ contributions

CWP conceived and designed the experiments, performed experiments, and analyzed data. QV and RW performed experiments and contributed to analysis of data. NC and SYY performed experiments. DEG conceived and designed experiments, analyzed data, and drafted the manuscript. FMD, SW, MPM, and MC contributed reagents, materials, and analysis tools. All authors read and approved the final manuscript.

## Supplementary Material

Additional file 1: Table S1 STS mapping primers. **Table S2.** Long range PCR and sequencing primers. **Table S3.** Frequency Of KIR Haplotypes in 4 ethnic groups. Click here for file

Additional file 2: Figure S1 Sequence analysis of new KIR haplotypes. Cartoons illustrating the gene content structures of recombinant haplotypes aligned with their putative parent haplotypes are shown with DNA sources and haplotype names respectively labeled. The extent of fosmid clones and long-range PCR products sequenced are indicated with green bars aligned with each set of cartoons. A color-coded key at the bottom indicates different types of gene rearrangements found in the new haplotypes. **Figure S2.** Phylogenetic and breakpoint analysis suggest possible recombination points in the centromeric and telomeric regions. (A) The 2DL23-2DS35 intergenic region and the exon 1-5 regions of 2DL2, 2DL3, and 2DS2 were subjected to phylogenetic analysis as described in Methods. Analysis of the 2DL23-2DS35 intergenic region revealed the presence of 4 major linkage groups and analysis of 2DL2, 2DL3, and 2DS2 sequences revealed 3 linkage groups, indicated by colored shading and labels. (B) Breakpoint analysis of three regions is presented. The pairwise identity plots indicate where crossover events are most likely to have occurred. Keys at the bottom of each plot identify the sequences that were used in the comparisons. Vertical bars at the top of each plot designate the positions of sequence differences. The exon-intron structures of the gene regions are indicated beneath each graph where appropriate with blue or pink designating the origins of the hybrid sequence. (C) Phylogenetic analysis was performed on two segments of KIR-3DL1 as indicated. The results for both regions are shown with the presence of the 2 major groups in blue (3DL1*015-like) or pink (3DL1*005-like). (D) Phylogenetic analysis was performed on three separate regions in KIR-3DL2 as indicated. Major groups of alleles are presented in green (3DL2*00701/*018), blue (3DL1*015-like) or pink (3DL1*005-like). Sequences and linkage information used in this analysis were from this study and previous haplotype sequencing [9]. **Figure S3.** (A) Identification of possible recombination events in the telomeric regions generating hybrid genes. Sequence alignments of 3DS1, 3DL1, and 3DL2 and the hybrid genes 3DS1/L1 and 3DL1/L2. In the sequence alignment plot, vertical bars represent SNP positions, color shading identifies the source gene, and the consensus gene structure is indicated beneath. Gene sequences are color-coded (3DS1, purple; 3DL1, blue; 3DL2 pink). We used full genomic sequence of 3DL1/L2 hybrid gene from EU267269. The orange shading represents a possible exchange segment between intron 5 and exon 6, flanked by L1PMA2 and MLT1D-like elements. Pairwise identity plots indicate where recombination events likely occurred. Keys at the bottom of each plot indicate the sequences that were used in the comparisons. Vertical bars at the top of each plot represent sequence differences. Haplotype motifs and exon-intron structures of 3DS1/3DL1 and 3DL1/3DL2 hybrid genes are shown. Color shading indicates the source content of the hybrid gene, aligned beneath the tA01-like motif within which they were identified. (B) Analysis of hybrid genes associated with KIR-2DL1, 2DS1, and 2DP1. Sequence alignment and breakpoint analysis comparing the genomic regions of 2DL1 (A/B), 2DS1, 2DP1 (A/B), and the hybrid genes 2DP1/L1 and 2DL1/S1. In the scale at the top, the green shading (darker) indicates the extent of sequence homology among the aligned sequences. A scaled cartoon of the consensus gene structure with repeat elements labeled is beneath the alignments. Two possible recombination sites in the intron 3 region indicated by red bars in the sequence alignment are supported by the sequence alignment and the pairwise identity plots. The patterns of mismatches (black hatches) indicate the positions of the minor allele SNP among the aligned sequences and above the pairwise identity graphs. Immediately beneath the graphs are color-coded keys identifying the sequences being analyzed. Breakpoint and phylogenetic analysis of the 2DL1A, 2DL1B, and 2DS1 sequences. The pairwise identity plot compares the sequences as indicated in the key, SNPs are represented by vertical bars above the graph, and the gene exon-intron structure is represented with boxes and lines at the bottom. Phylogenetic analysis distinguished three groups as indicated by color shading. Modeling of recombination between 2DL1, 2DS1, and 2DP1 in the intron 3 region. Consequent known hybrid genes are depicted as are hybrid genes not yet identified (boxed). (C) Putative recombination between the 2DL5 and 3DP1 genes is localized to the intron 2 regions. Sequence alignments of the genomic segment from exon 1 to intron 2 in KIR2DL5 and KIR3DP1 are shown. Source DNAs in the alignment are identified on the left and the structures of the recombinant segments are drawn beneath the alignment. The color-coded key at the bottom of the alignment indicates the origin of respective sequences. Breakpoint analysis revealed a likely recombination site in the intron 2 region. Pairwise identity plots of the 2DL5A, 2DL5B, and 3DP1 sequences are shown. The patterns of mismatches (black hatches) indicate the positions of the minor allele SNP among the aligned sequences and coloring indicating the sequence origin according to the key. KIR2DL5 was divided into two regions (exon 1 to exon 2 and intron 2 to exon 9) by phylogenetic analysis. Analysis of the exon 1-exon 2 region revealed two groups, 2DL5A (telomeric; blue) and 2DL5B (centromeric; pink). Analysis of the second region, intron 3 to exon 9, also revealed two groups, 2DL5-2DS3 (peach) and 2DL5-2DS5 (green). **Figure S4.** Putative recombination between the 2DL5 and 3DP1 genes is localized to the intron 2 regions. (A) Sequence alignments of the genomic segment from exon 1 to intron 2 in KIR2DL5 and KIR3DP1 are shown. Source DNAs in the alignment are identified on the left and the structures of the recombinant segments are drawn beneath the alignment. The color-coded key at the bottom of the alignment indicates the origin of respective sequences. (B) Breakpoint analysis revealed a likely recombination site in the intron 2 region. Pairwise identity plots of the 2DL5A, 2DL5B, and 3DP1 sequences are shown. The patterns of mismatches (black hatches) indicate the positions of the minor allele SNP among the aligned sequences and coloring indicating the sequence origin according to the key. (C) KIR2DL5 was divided into two regions (exon 1 to exon 2 and intron 2 to exon 9) by phylogenetic analysis. Analysis of the exon 1-exon 2 region revealed two groups, 2DL5A (telomeric; blue) and 2DL5B (centromeric; pink). Analysis of the second region, intron 3 to exon 9, also revealed two groups, 2DL5-2DS3 (peach) and 2DL5-2DS5 (green). **Figure S5.** Analysis of hybrid genes associated with KIR-2DL1, 2DS1, and 2DP1. (A) Sequence alignment and breakpoint analysis comparing the genomic regions of 2DL1 (cA01 motif/cB01 motif), 2DS1, 2DP1 (cA01 motif/cB01 motif), and the hybrid genes 2DP1/L1 and 2DL1/S1. In the scale at the top, the green shading (darker) indicates the extent of sequence homology among the aligned sequences. A scaled cartoon of the consensus gene structure with repeat elements labeled is beneath the alignments. Two possible recombination sites in the intron 3 region (red bars) are supported by the sequence alignment and the pairwise identity plots. The patterns of mismatches (black hatches) indicate the positions of the minor allele SNP among the aligned sequences and above the pairwise identity graphs. Immediately beneath the graphs are color-coded keys identifying the sequences being analyzed. (B) Breakpoint and phylogenetic analysis of the 2DL1A, 2DL1B, and 2DS1 sequences. The pairwise identity plot compares the sequences as indicated in the key, SNPs are represented by vertical bars above the graph, and the gene exon-intron structure is represented with boxes and lines at the bottom. Phylogenetic analysis distinguished three groups as indicated by color shading. (C) Modeling of recombination between 2DL1, 2DS1, and 2DP1 in the intron 3 region. Boxed genes are hybrid genes not yet identified. **Figure S6.** Peak ratio determines copy number for KIR haplotype analysis. Examples of ABI chromatograms are presented showing peak ratio differences from sequence analysis of assay 2DS1-4-002. This assay amplifies homologous sequences from three different loci as described in methods, with a key base distinguishing each locus at position 257. The five examples show different copy number ratios for 2DS1 (A):2DL1 (C):2DS4 (T) as indicated beneath each chromatogram. The copy number was validated from control samples where the complete phased genomic sequences from both haplotypes were available. (PDF 2241 kb) Click here for file
