# Supplementary material for: Recombinant structures expand and contract inter and intragenic diversification at the KIR locus
Source: BMC Genomics. 2013 Feb 8;14:89. doi: 10.1186/1471-2164-14-89 (PMC3606631; doi:10.1186/1471-2164-14-89)
Supplement: Additional file 1: Table S1 — STS mapping primers. Table S2. Long range PCR and sequencing primers. Table S3. Frequency Of KIR Haplotypes in 4 ethnic groups. [file 1471-2164-14-89-S1.pdf]

Table S1. STS mapping primers.

Gene		*Primer sequence*	*Position*	*Size*
3DL3	F	CACAGAAAACCTTCCCTCCTGGC	Exon 4	216
	R	GGTCCCTGCAAGGGCATGTG **(G)**	Exon 4	
2DS2	F	GCACTTCCTTCTGCACAGAGAGGGGAACTA **(G)**	Exon 4	191
	R	TGTCCAGAGGGTCACTGGGAGC	Exon 4	
2DL3	F	**CTTCCAG**GTAACCCCAGACACCTGCAT	Exon 7	132
	R	**CTCTGCTTCGTAAGACTTAC**TTTTTTTGTCGC **(T)**	Exon 7	
2DL2	F	CTCCTGGCCCACCCAGGACG **(T)**	Exon 4	196
	R	CTGCAAGGTCTTGCATCACGG **(T)**	Exon 4	
2DL5	F	GACCAAGAGCCTGCGGGGG **(C)**	Exon 8	315
	R	TGCTTATGGGCAGGAGACAATGAT	Exon 9	
2DL5.2	F	TGTAAATCAATATCTGGCAGAGGAGTGGTA **(A)**	Intron 6	365
	R	GACCCCGCCAAACCTCACG	Intron 6	
2DL5.1	F	AATCAATATCTGGCAGAGGAGTGGTT **(A)**	Intron 6	361
	R	GACCCCGCCAAACCTCACG	Intron 6	
2DS3	F	ACTTTGCGCCTCATTGGAGAGCACAT	Exon 4	157
	R	CGATGTCCAGAGGGTCACTGGGAGCTCAA **(G)**	Exon 4	
2DS3/5	F	CGCCTCATTGGAGAGCACAT	Exon 4	148
	R	TGTCCAGAGGGTCACTGGGAGC	Exon 4	
2DP1 including exon3	F	CACAGAAAACCTTCCCTCCTGGC	Exon 4	270
	R	CACTGGGAGCTGACAACTGCTG **(A)**	Exon 4	
2DP1_hpltA including exon3	F	CACAGAAAACCTTCCCTCCTGGC	Exon 4	187
	R	CGATGGAGAAGTTGGCCTTGTAA **(G)**	Exon 4	
2DP1_hpltB including exon3	F	CTTCTGCACAGAGAGGGGCCA **(A)**	Exon 4	183
	R	TGTCCAGAGGGTCACTGGGAGC	Exon 4	
2DL1	F	TCCAAGGCCAACTTCTCCAACA **(T)**	Exon 4	113
	R	AGAGGGTCACTGGGAGCTGAAAC **(C)**	Exon 4	
3DP1	F	TCTGCACAGAGAGGGGAAGTTTCAT **(A)**	Exon 4	182
	R	TGTCCAGAGGGTCACTGGGAGC	Exon 4	
2DL4A	F	CGGGCCCCACGGTTCACA **(G)**	Exon 5	171
	R	GGGTGGCAGGACCCAGAGG	Exon 5	
2DL4B	F	GGGCCCCACGGTTCACG **(G)**	Exon 5	170
	R	GGGTGGCAGGACCCAGAGG	Exon 5	
3DL1A	F	TGCCTGGCCCAGCGCTGTGGT	Exon 3	154
	R	GCTCATGTTGAAGCTCTCCTGGAAAAA **(T)**	Exon 3	
3DL1B	F	TGCCTGGCCCAGCGCTGTGGT	Exon 3	133
	R	GAATATTCTGCCATGGAAGATGGGGAT **(A)**	Exon 3	
2DS5	F	TTGCGCCTCATTGGAGAGCACAT	Exon 4	147
	R	CAGAGGGTCACTGGGCGCTCAC **(G)**	Exon 4	
3DS1	F	AAGAGCCTGCAGGGAACAGAAGT	Exon 8	214
	R	GGGCGAGTGATTTTTCTCTGTGTGA	Exon 9	
2DS4L	F	GCTCCTATGACATGTACCATCTATCCA	Exon 5	177
	R	AGTTTGACCACTCGTAGGGGGC **(A)**	Exon 5	
2DS4S deletion from (003, 004, 006, 007 and 009)	F	TGTCCTGCAGCTCCATCTATCCA	Exon 5	173
	R	AGTTTGACCACTCGTAGGGGGC **(A)**	Exon 5	
2DS1	F	CCAACTTCTCCATCAGTCGCATCAA **(G)**	Exon 4	105
	R	GAGGGTCACTGGGAGCTGAGAA **(C)**	Exon 4	
3DL2	F	GCCCCACGGTTCAGGCAGG	Exon 5	222
	R	ACCACACGCAGGGCAGGG	Exon 5	

Sequence modification for specificity on PCR; Original sequence; Intron sequence

Table S2. Long range PCR and sequencing primers.

Gene		*Primer sequence*
3DL2-Fcar	F	GTAAGCACAGAATTCAATCACCTCATGTG
	R	TTTTCCTTCCACAACAATCACAGTTTT
3DL3 (exon7)-2DL2 (exon5)	F	TCAGTGGTCATCATCCCCTTTGCT
	R	GTCTTCTCTCTGCATCTGTCCATGCTTA
3DS1-3DL2 hybrid	F	TTCTAGTGAGAGCAATTTCCAGGAAGCCATGC
	R	GCTAAGCAAAGGAGTGTGTTTTCTT
3DL1-3DL2 hybrid	F	GACGCGAGGTGTCAATTCTAGTGAGAG
	R	GGTGGGCAGGGGTCAAGTGAAATAGATAC
2DL4	F	GACGTAGAAGAAGCCTACCTATGTCCCCTTCA
	R	AGGCACCAGATTTGTGGTGTGAGGAAGAGT
3DL1-exon3-sequencing	F	CCAGGTGTGGTAGGAGCCTTAGAAA
	R	TTCAATCTCCCTTGACCCCAAATA
3DL1-exon4-sequencing	F	CATGGATGGGATGATAAAGAGAGA
	R	GAATCCCACACTTATCTTCCTCATG
3DL1-exon5-sequencing	F	AGGGGAGTGAGTTCTCAGCTCAGGT
	R	GCTAAGGATTTAGGATCATAGGACATG
3DL2-exon7-sequencing	F	AAGCTGGGTCTCCCGCCATCAGGC
	R	AGAAAGCCCTGCCTCTGTGGCTCCTC
3DL2-exon89-sequencing	F	TGAGTCTGGATGTTGGCAGCTGAAGA
	R	ACTGATGCCTTCAGATTCCAGCTGCTGGTT

Table S3. Frequency of KIR haplotypes in 4 ethnic groups.

Haplotype	AFA (%)		ASI (%)		HIS (%)		CAU (%)	
cA01|tA01	51	(53.1)	58	(63.0)	57	(60.6)	57	(62.0)
cA01|tB01	4	(4.2)	16	(17.4)	16	(17.0)	10	(10.9)
cB01|tB01	2	(2.1)	1	(1.1)			4	(4.3)
cB02|tA01	6	(6.3)	4	(4.3)	8	(8.5)	14	(15.2)
cB01|tA01	15	(15.6)	3	(3.3)	8	(8.5)	5	(5.4)
cB02|tB01	2	(2.1)	5	(5.4)	3	(3.2)	1	(1.1)
cB03|tA01	2	(2.1)						
cA01|tB01-del7/8			1	(1.1)				
cA01|tA01-hybd1	4	(4.2)						
cA01|tA01-ins3	2	(2.1)						
cA01|tA01-ins5							1	(1.1)
cB01|tB01-del6	1	(1.0)						
cB02|tA01-hybd1	1	(1.0)						
cB01|tA01-del3	2	(2.1)						
cB01|tA01-ins4			1	(1.1)				
cB01|tA01-hybd1	2	(2.1)						
cB01|tA01-hybd2	1	(1.0)						
cB02|tB01-del6	1	(1.0)	3	(3.3)	2	(2.1)		
