# Supplementary material for: Recombinant structures expand and contract inter and intragenic diversification at the KIR locus
Source: BMC Genomics. 2013 Feb 8;14:89. doi: 10.1186/1471-2164-14-89 (PMC3606631; doi:10.1186/1471-2164-14-89)
Supplement: Additional file 2: Figure S1 — Sequence analysis of new KIR haplotypes. Cartoons illustrating the gene content structures of recombinant haplotypes aligned with their putative parent haplotypes are shown with DNA sources and haplotype names respectively labeled. The extent of fosmid clones and long-range PCR products sequenced are indicated with green bars aligned with each set of cartoons. A color-coded key at the bottom indicates different types of gene rearrangements found in the new haplotypes. Figure S2. Phylogenetic and breakpoint analysis suggest possible recombination points in the centromeric and telomeric regions. (A) The 2DL23-2DS35 intergenic region and the exon 1-5 regions of 2DL2, 2DL3, and 2DS2 were subjected to phylogenetic analysis as described in Methods. Analysis of the 2DL23-2DS35 intergenic region revealed the presence of 4 major linkage groups and analysis of 2DL2, 2DL3, and 2DS2 sequences revealed 3 linkage groups, indicated by colored shading and labels. (B) Breakpoint analysis of three regions is presented. The pairwise identity plots indicate where crossover events are most likely to have occurred. Keys at the bottom of each plot identify the sequences that were used in the comparisons. Vertical bars at the top of each plot designate the positions of sequence differences. The exon-intron structures of the gene regions are indicated beneath each graph where appropriate with blue or pink designating the origins of the hybrid sequence. (C) Phylogenetic analysis was performed on two segments of KIR-3DL1 as indicated. The results for both regions are shown with the presence of the 2 major groups in blue (3DL1*015-like) or pink (3DL1*005-like). (D) Phylogenetic analysis was performed on three separate regions in KIR-3DL2 as indicated. Major groups of alleles are presented in green (3DL2*00701/*018), blue (3DL1*015-like) or pink (3DL1*005-like). Sequences and linkage information used in this analysis were from this study and previous haplotype sequencing [9]. Figure S3. (A) Ide [file 1471-2164-14-89-S2.pdf]

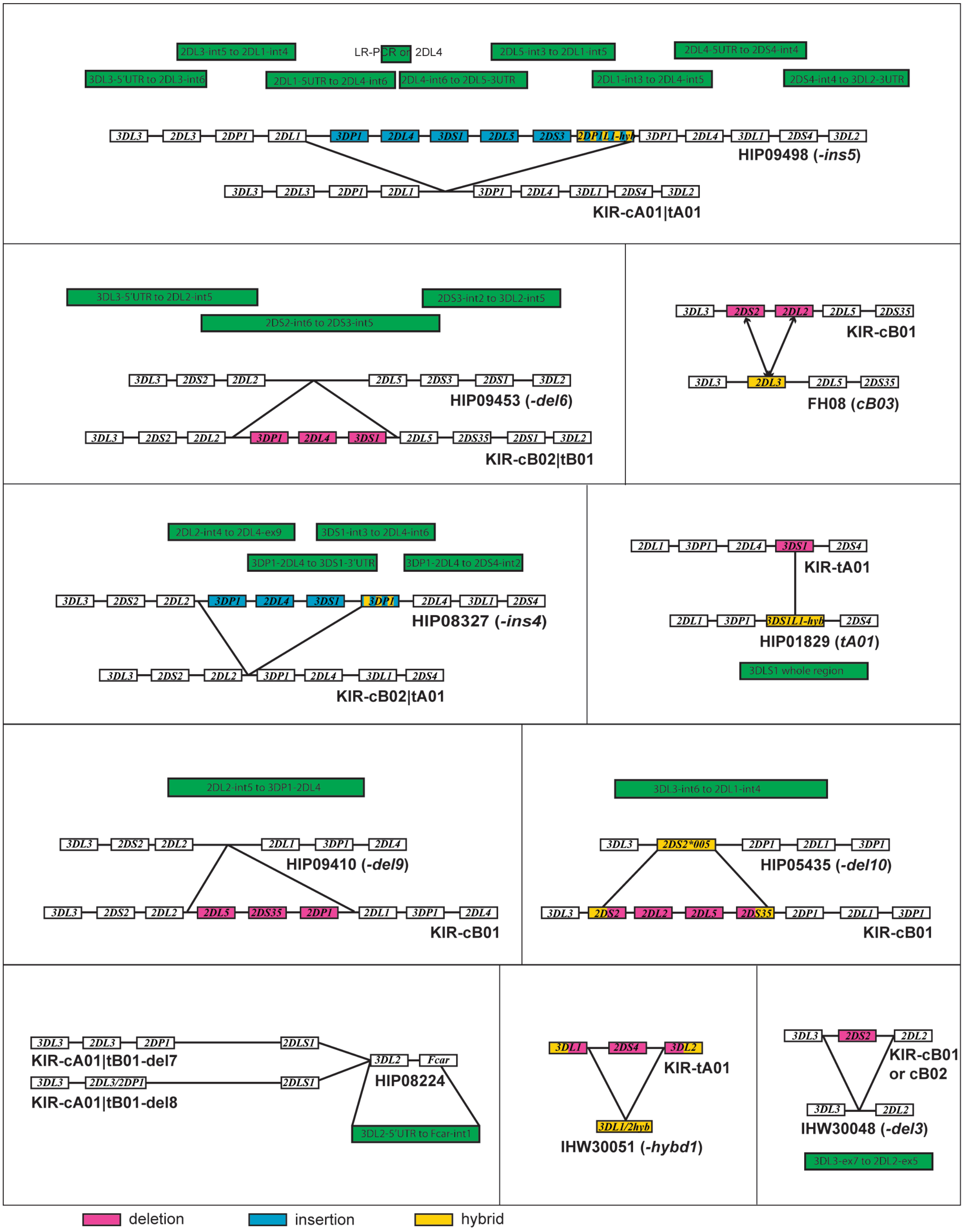

Figure S1. Sequence analysis of new KIR haplotypes. Cartoons illustrating the gene content structures of recombinant haplotypes aligned with their putative parent haplotypes are shown with DNA sources and haplotype names respectively labeled. The extent of fosmid clones and long-range PCR products sequenced are indicated with green bars aligned with each set of cartoons. A color-coded key at the bottom indicates different types of gene rearrangements found in the new haplotypes.

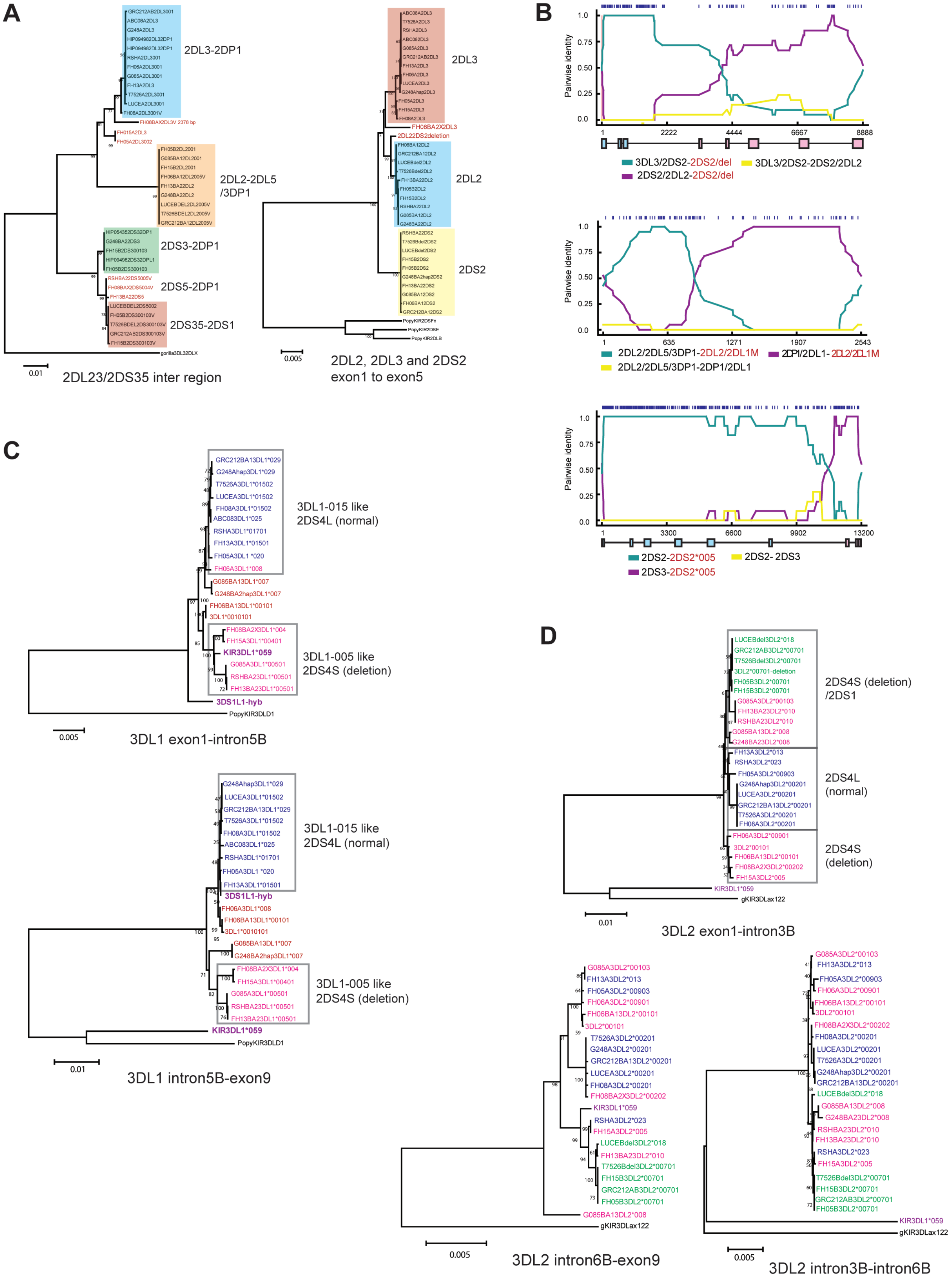

A
2DL3-2DP1
2DL2-2DL5
/3DP1
2DS3-2DP1
2DS5-2DP1
2DS35-2DS1
0.01
2DL23/2DS35 inter region
2DL3
2DL2
2DS2
0.005
2DL2, 2DL3 and 2DS2
exon1 to exon5
B
Pairwise identity
1.0
0.75
0.50
0.25
0.0
1
2222
4444
6667
8888
3DL3/2DS2-2DS2/del
3DL3/2DS2-2DS2/2DL2
2DS2/2DL2-2DS2/del
635
1271
1907
2543
2DL2/2DL5/3DP1-2DL2/2DL1M
2DP1/2DL1-2DL2/2DL1M
2DL2/2DL5/3DP1-2DP1/2DL1
3300
6600
9902
13200
2DS2-2DS2*005
2DS2- 2DS3
2DS3-2DS2*005
C
GRC212BA13DL1*029
G248Ahap3DL1*029
T7526A3DL1*01502
LUCEA3DL1*01502
FH08A3DL1*01502
ABC083DL1*025
RSHA3DL1*01701
FH13A3DL1*01501
FH05A3DL1 *020
FH06A3DL1*008
G085BA13DL1*007
G248BA2hap3DL1*007
FH06BA13DL1*00101
3DL1*0010101
FH08BA2X3DL1*004
FH15A3DL1*00401
KIR3DL1*059
G085A3DL1*00501
RSHBA23DL1*00501
FH13BA23DL1*00501
3DS1L1-hyb
PopyKIR3DLD1
3DL1-015 like
2DS4L (normal)
3DL1-005 like
2DS4S (deletion)
0.005
3DL1 exon1-intron5B
G248Ahap3DL1*029
LUCEA3DL1*01502
GRC212BA13DL1*029
T7526A3DL1*01502
FH08A3DL1*01502
ABC083DL1*025
RSHA3DL1*01701
FH05A3DL1 *020
FH13A3DL1*01501
3DS1L1-hyb
FH06A3DL1*008
FH06BA13DL1*00101
3DL1*0010101
G085BA13DL1*007
G248BA2hap3DL1*007
FH08BA2X3DL1*004
FH15A3DL1*00401
G085A3DL1*00501
RSHBA23DL1*00501
FH13BA23DL1*00501
KIR3DL1*059
PopyKIR3DLD1
3DL1-015 like
2DS4L (normal)
3DL1-005 like
2DS4S (deletion)
0.01
3DL1 intron5B-exon9
D
LUCEBdel3DL2*018
GRC212AB3DL2*00701
T7526Bdel3DL2*00701
3DL2*00701-deletion
FH05B3DL2*00701
FH15B3DL2*00701
G085A3DL2*00103
FH13BA23DL2*010
RSHBA23DL2*010
G085BA13DL2*008
G248BA23DL2*008
FH13A3DL2*013
RSHA3DL2*023
FH05A3DL2*00903
G248Ahap3DL2*00201
LUCEA3DL2*00201
GRC212BA13DL2*00201
T7526A3DL2*00201
FH08A3DL2*00201
FH06A3DL2*00901
3DL2*00101
FH06BA13DL2*00101
FH08BA2X3DL2*00202
FH15A3DL2*005
KIR3DL1*059
gKIR3DLax122
2DS4S (deletion)
/2DS1
2DS4L
(normal)
2DS4S
(deletion)
0.01
3DL2 exon1-intron3B
0.005
3DL2 intron6B-exon9
0.005
3DL2 intron3B-intron6B

Figure S2. Phylogenetic and breakpoint analysis suggest possible recombination points in the centromeric and telomeric regions. (A) The 2DL23-2DS35 intergenic region and the exon 1-5 regions of 2DL2, 2DL3, and 2DS2 were subjected to phylogenetic analysis as described in Methods. Analysis of the 2DL23-2DS35 intergenic region revealed the presence of 4 major linkage groups and analysis of 2DL2, 2DL3, and 2DS2 sequences revealed 3 linkage groups, indicated by colored shading and labels. (B) Breakpoint analysis of three regions is presented. The pairwise identity plots indicate where crossover events are most likely to have occurred. Keys at the bottom of each plot identify the sequences that were used in the comparisons. Vertical bars at the top of each plot designate the positions of sequence differences. The exon-intron structures of the gene regions are indicated beneath each graph where appropriate with blue or pink designating the origins of the hybrid sequence. (C) Phylogenetic analysis was performed on two segments of KIR-3DL1 as indicated. The results for both regions are shown with the presence of the 2 major groups in blue (3DL1*015-like) or pink (3DL1*005-like). (D) Phylogenetic analysis was performed on three separate regions in KIR-3DL2 as indicated. Major groups of alleles are presented in green (3DL2*00701/*018), blue (3DL1*015-like) or pink (3DL1*005-like). Sequences and linkage information used in this analysis were from this study and previous haplotype sequencing [9].

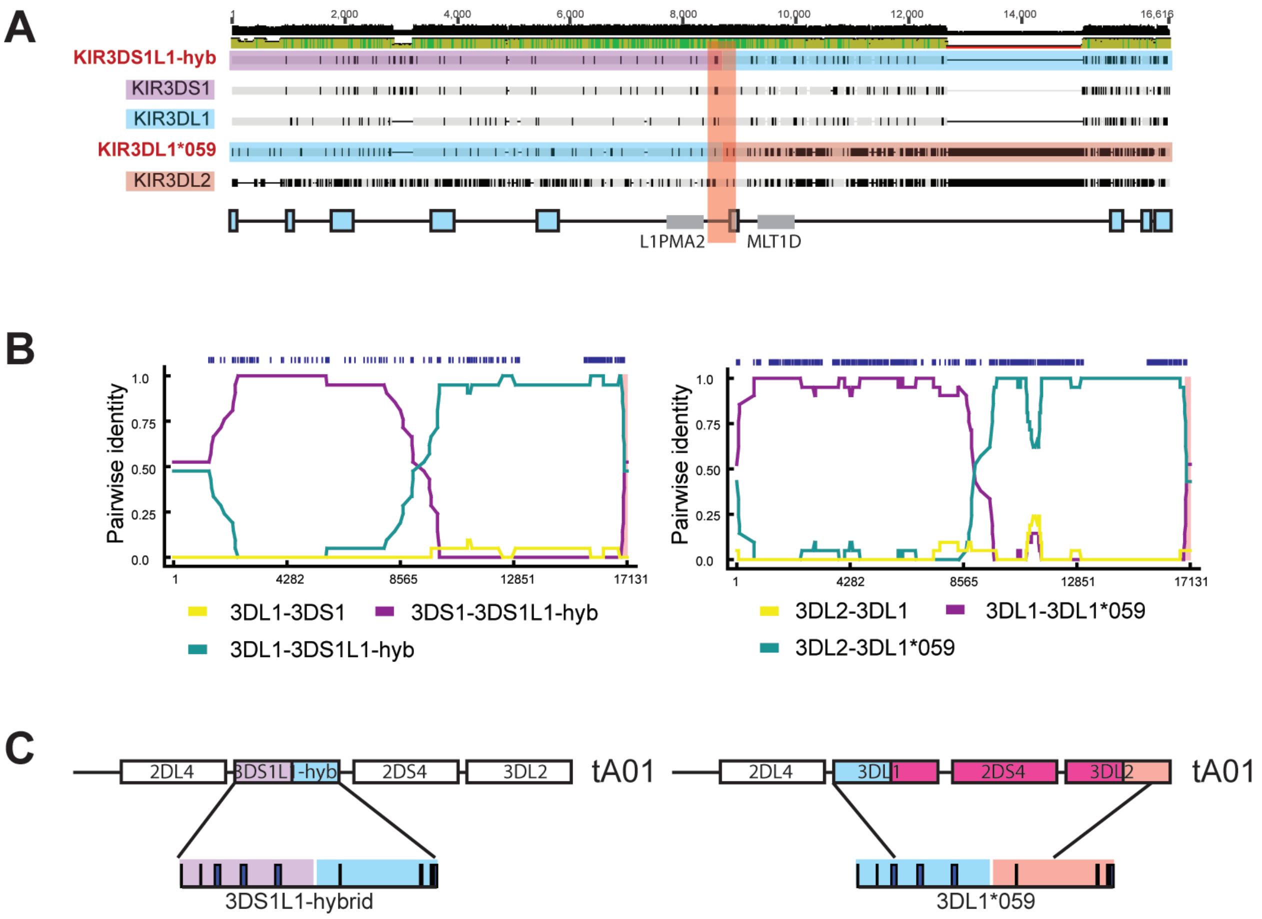

Figure S3. (A) Identification of possible recombination events in the telomeric regions generating hybrid genes. Sequence alignments of 3DS1, 3DL1, and 3DL2 and the hybrid genes 3DS1/L1 and 3DL1/L2. In the sequence alignment plot, vertical bars represent SNP positions, color shading identifies the source gene, and the consensus gene structure is indicated beneath. Gene sequences are color-coded (3DS1, purple; 3DL1, blue; 3DL2 pink). We used full genomic sequence of 3DL1/L2 hybrid gene from EU267269. The orange shading represents a possible exchange segment between intron 5 and exon 6, flanked by L1PMA2 and MLT1D-like elements. Pairwise identity plots indicate where recombination events likely occurred. Keys at the bottom of each plot indicate the sequences that were used in the comparisons. Vertical bars at the top of each plot represent sequence differences. Haplotype motifs and exon-intron structures of 3DS1/3DL1 and 3DL1/3DL2 hybrid genes are shown. Color shading indicates the source content of the hybrid gene, aligned beneath the tA01-like motif within which they were identified. **(B)** Analysis of hybrid genes associated with KIR-2DL1, 2DS1, and 2DP1. Sequence alignment and breakpoint analysis comparing the genomic regions of 2DL1 (A/B), 2DS1, 2DP1 (A/B), and the hybrid genes 2DP1/L1 and 2DL1/S1. In the scale at the top, the green shading (darker) indicates the extent of sequence homology among the aligned sequences. A scaled cartoon of the consensus gene structure with repeat elements labeled is beneath the alignments. Two possible recombination sites in the intron 3 region indicated by red bars in the sequence alignment are supported by the sequence alignment and the pairwise identity plots. The patterns of mismatches (black hatches) indicate the positions of the minor allele SNP among the aligned sequences and above the pairwise identity graphs. Immediately beneath the graphs are color-coded keys identifying the sequences being analyzed. Breakpoint and phylogenetic analysis of the 2DL1A, 2DL1B, and 2DS1

sequences. The pairwise identity plot compares the sequences as indicated in the key, SNPs are represented by vertical bars above the graph, and the gene exon-intron structure is represented with boxes and lines at the bottom. Phylogenetic analysis distinguished three groups as indicated by color shading. Modeling of recombination between 2DL1, 2DS1, and 2DP1 in the intron 3 region. Consequent known hybrid genes are depicted as are hybrid genes not yet identified (boxed). **(C)** Putative recombination between the 2DL5 and 3DP1 genes is localized to the intron 2 regions. Sequence alignments of the genomic segment from exon 1 to intron 2 in KIR2DL5 and KIR3DP1 are shown. Source DNAs in the alignment are identified on the left and the structures of the recombinant segments are drawn beneath the alignment. The color-coded key at the bottom of the alignment indicates the origin of respective sequences. Breakpoint analysis revealed a likely recombination site in the intron 2 region. Pairwise identity plots of the 2DL5A, 2DL5B, and 3DP1 sequences are shown. The patterns of mismatches (black hatches) indicate the positions of the minor allele SNP among the aligned sequences and coloring indicating the sequence origin according to the key. KIR2DL5 was divided into two regions (exon 1 to exon 2 and intron 2 to exon 9) by phylogenetic analysis. Analysis of the exon 1-exon 2 region revealed two groups, 2DL5A (telomeric; blue) and 2DL5B (centromeric; pink). Analysis of the second region, intron 3 to exon 9, also revealed two groups, 2DL5-2DS3 (peach) and 2DL5-2DS5 (green).

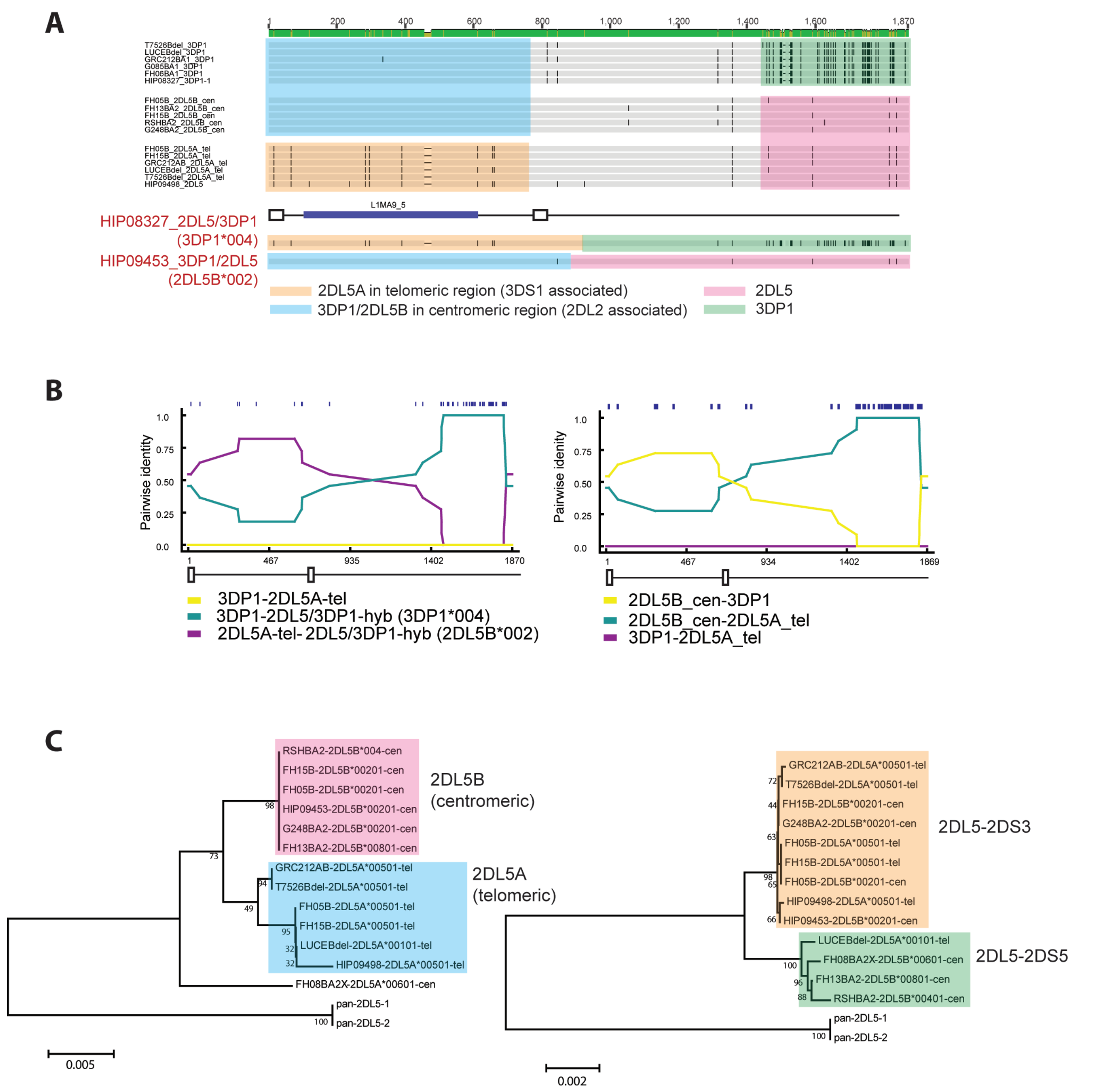

Figure S4. Putative recombination between the 2DL5 and 3DP1 genes is localized to the intron 2 regions. (A) Sequence alignments of the genomic segment from exon 1 to intron 2 in KIR2DL5 and KIR3DP1 are shown. Source DNAs in the alignment are identified on the left and the structures of the recombinant segments are drawn beneath the alignment. The color-coded key at the bottom of the alignment indicates the origin of respective sequences. (B) Breakpoint analysis revealed a likely recombination site in the intron 2 region. Pairwise identity plots of the 2DL5A, 2DL5B, and 3DP1 sequences are shown. The patterns of mismatches (black hatches) indicate the positions of the minor allele SNP among the aligned sequences and coloring indicating the sequence origin according to the key. (C) KIR2DL5 was divided into two regions (exon 1 to exon 2 and intron 2 to exon 9) by phylogenetic analysis. Analysis of the exon 1-exon 2 region revealed two groups, 2DL5A (telomeric; blue) and 2DL5B (centromeric; pink). Analysis of the second region, intron 3 to exon 9, also revealed two groups, 2DL5-2DS3 (peach) and 2DL5-2DS5 (green).

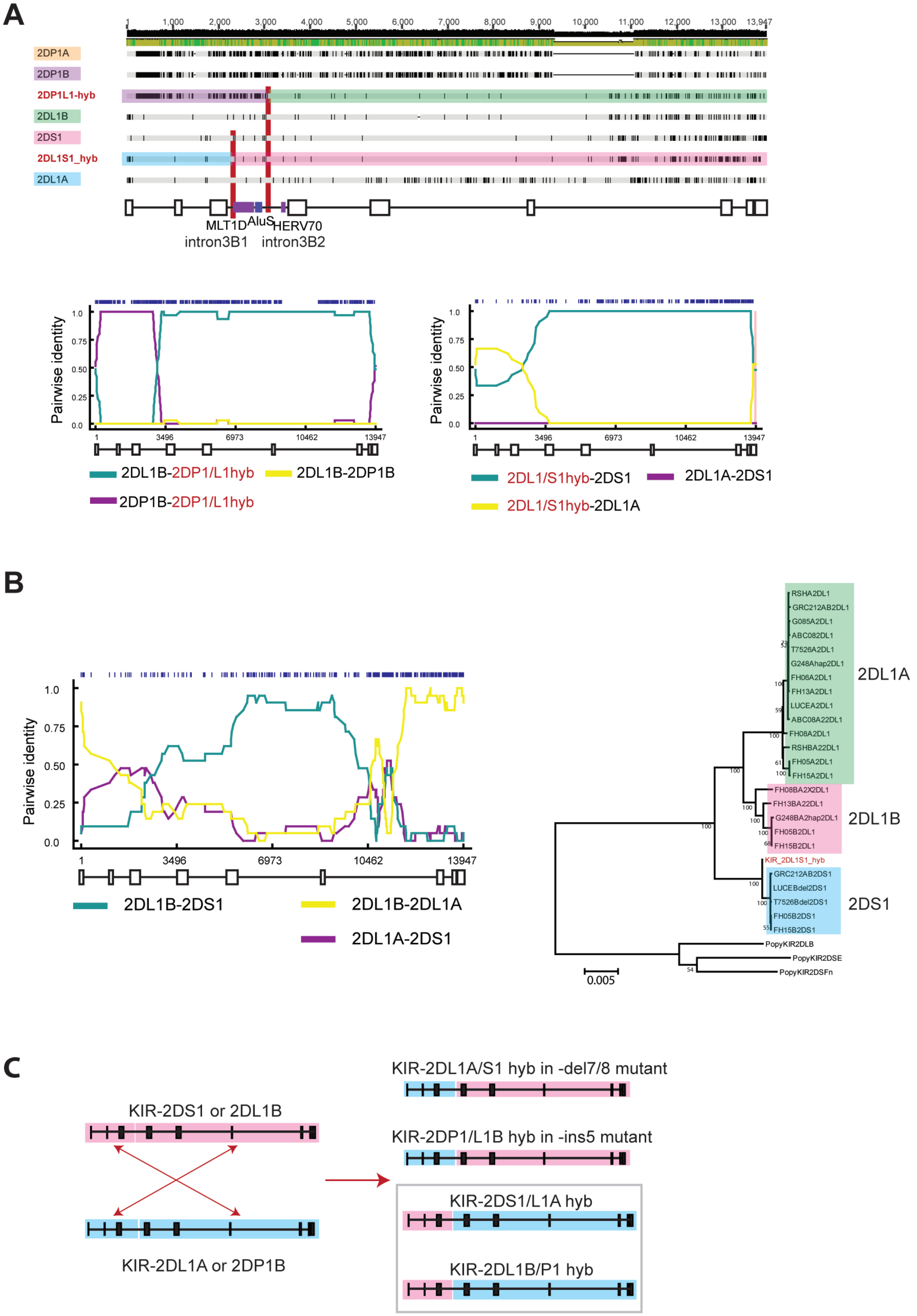

A
2DP1A
2DP1B
2DP1L1-hyb
2DL1B
2DS1
2DL1S1_hyb
2DL1A
MLT1D
AluS
HERV70
intron3B1
intron3B2
Pairwise identity
2DL1B-2DP1/L1hyb
2DL1B-2DP1B
2DP1B-2DP1/L1hyb
2DL1/S1hyb-2DS1
2DL1A-2DS1
2DL1/S1hyb-2DL1A
B
Pairwise identity
2DL1B-2DS1
2DL1B-2DL1A
2DL1A-2DS1
2DL1A
2DL1B
2DS1
KIR_2DL1S1_hyb
PopyKIR2DLB
PopyKIR2DSE
PopyKIR2DSFn
0.005
C
KIR-2DS1 or 2DL1B
KIR-2DL1A or 2DP1B
KIR-2DL1A/S1 hyb in -del7/8 mutant
KIR-2DP1/L1B hyb in -ins5 mutant
KIR-2DS1/L1A hyb
KIR-2DL1B/P1 hyb

Figure S5. Analysis of hybrid genes associated with KIR-2DL1, 2DS1, and 2DP1. (A) Sequence alignment and breakpoint analysis comparing the genomic regions of 2DL1 (cA01 motif/cB01 motif), 2DS1, 2DP1 (cA01 motif/cB01 motif), and the hybrid genes 2DP1/L1 and 2DL1/S1. In the scale at the top, the green shading (darker) indicates the extent of sequence homology among the aligned sequences. A scaled cartoon of the consensus gene structure with repeat elements labeled is beneath the alignments. Two possible recombination sites in the intron 3 region (red bars) are supported by the sequence alignment and the pairwise identity plots. The patterns of mismatches (black hatches) indicate the positions of the minor allele SNP among the aligned sequences and above the pairwise identity graphs. Immediately beneath the graphs are color-coded keys identifying the sequences being analyzed. (B) Breakpoint and phylogenetic analysis of the 2DL1A, 2DL1B, and 2DS1 sequences. The pairwise identity plot compares the sequences as indicated in the key, SNPs are represented by vertical bars above the graph, and the gene exon-intron structure is represented with boxes and lines at the bottom. Phylogenetic analysis distinguished three groups as indicated by color shading. (C) Modeling of recombination between 2DL1, 2DS1, and 2DP1 in the intron 3 region. Boxed genes are hybrid genes not yet identified.

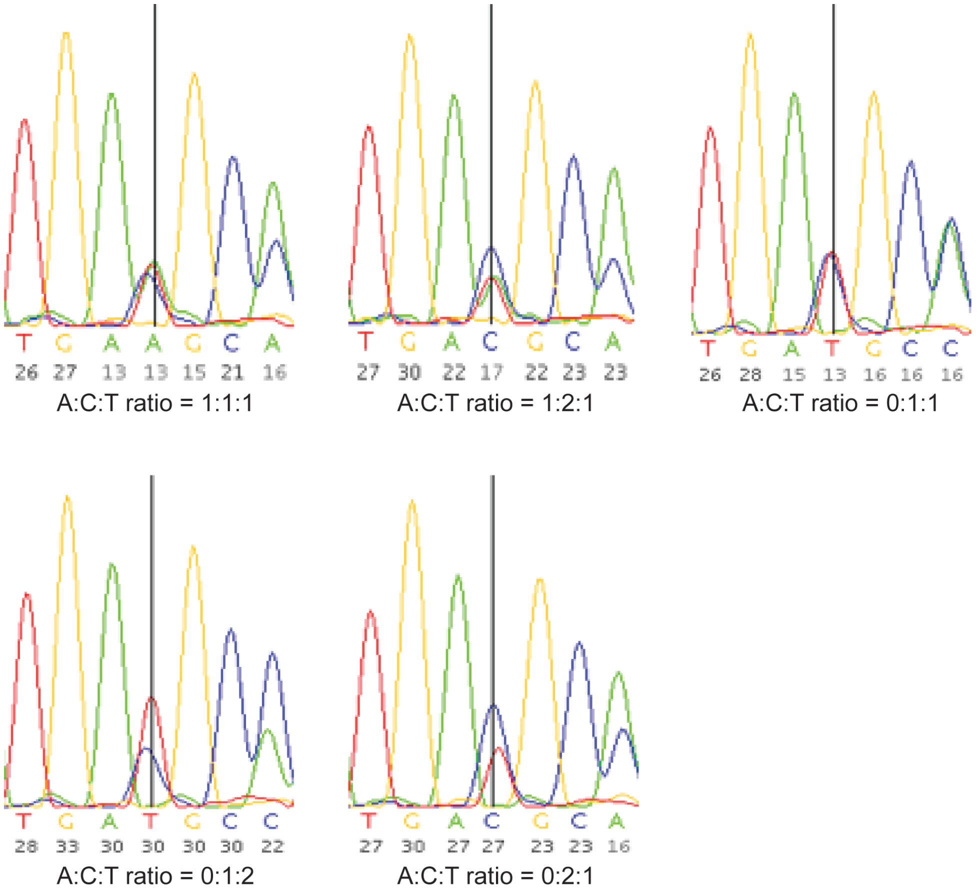

Figure S6. Peak ratio determines copy number for KIR haplotype analysis. Examples of ABI chromatograms are presented showing peak ratio differences from sequence analysis of assay 2DS1-4-002. This assay amplifies homologous sequences from three different loci as described in methods, with a key base distinguishing each locus at position 257. The five examples show different copy number ratios for 2DS1 (A):2DL1 (C):2DS4 (T) as indicated beneath each chromatogram. The copy number was validated from control samples where the complete phased genomic sequences from both haplotypes were available.
